# Identification of New Peptides from Fermented Milk Showing Antioxidant Properties: Mechanism of Action

**DOI:** 10.3390/antiox9020117

**Published:** 2020-01-29

**Authors:** Federica Tonolo, Federico Fiorese, Laura Moretto, Alessandra Folda, Valeria Scalcon, Alessandro Grinzato, Stefania Ferro, Giorgio Arrigoni, Alberto Bindoli, Emiliano Feller, Marco Bellamio, Oriano Marin, Maria Pia Rigobello

**Affiliations:** 1 Department of Biomedical Sciences, University of Padova, 35131 Padova, Italy; federica.tonolo@phd.unipd.it (F.T.); federico.fiorese.1@gmail.com (F.F.); laura.moretto@unipd.it (L.M.); alessandra.folda.1@unipd.it (A.F.); valeria.scalcon@unipd.it (V.S.); alessandro.grinzato@phd.unipd.it (A.G.); stefania.ferro.1@unipd.it (S.F.); giorgio.arrigoni@unipd.it (G.A.); 2 Institute of Neuroscience, CNR, 35131 Padova, Italy; alberto.bindoli@bio.unipd.it; 3 Centrale del Latte di Vicenza S.p.A., 36100 Vicenza, Italy; feller@centralelatte.vicenza.it (E.F.); bellamio@centralelatte.vicenza.it (M.B.)

**Keywords:** bioactive peptides, Keap1/Nrf2 pathway, natural antioxidants, oxidative stress

## Abstract

Due to their beneficial properties, fermented foods are considered important constituents of the human diet. They also contain bioactive peptides, health-promoting compounds studied for a wide range of effects. In this work, several antioxidant peptides extracted from fermented milk proteins were investigated. First, enriched peptide fractions were purified and analysed for their antioxidant capacity in vitro and in a cellular model. Subsequently, from the most active fractions, 23 peptides were identified by mass spectrometry MS/MS), synthesized and tested. Peptides **N-15-M**, **E-11-F**, **Q-14-R** and **A-17-E** were selected for their antioxidant effects on Caco-2 cells both in the protection against oxidative stress and inhibition of ROS production. To define their action mechanism, the activation of the Kelch-like ECH-associated protein 1/nuclear factor erythroid 2-related factor 2(Keap1/Nrf2) pathway was studied evaluating the translocation of Nrf2 from cytosol to nucleus. In cells treated with **N-15-M**, **Q-14-R** and **A-17-E,** a higher amount of Nrf2 was found in the nucleus with respect to the control. In addition, the three active peptides, through the activation of Keap1/Nrf2 pathway, led to overexpression and increased activity of antioxidant enzymes. Molecular docking analysis confirmed the potential ability of **N-15-M**, **Q-14-R** and **A-17-E** to bind Keap1, showing their destabilizing effect on Keap1/Nrf2 interaction.

## 1. Introduction

In order to increase the shelf life and improve the flavour, fermentation is a process used for many years in a wide variety of food matrices such as soy, milk, meat and vegetables. Due to the presence of health-promoting compounds, fermented foods are considered important components of the human diet [1]. In fact, this type of food processing not only improves the organoleptic properties of the food matrix, but it can also increase the availability of various constituents, which can exert positive effects after consumption [2]. Among these compounds, bioactive peptides are inactive in the primary structure of proteins and can be released from them by fermenting bacteria-mediated proteolysis [3]. Many microorganisms are utilized in this process and it is well known that different fermenting strains can generate various patterns of bioactive peptides [4,5,6]. In particular, for dairy products, *Streptococcus thermophilus*, *Lactobacillus delbrueckii* subsp. *bulgaricus* and *Bifidobacterium* spp are usually employed [2,7]. Bioactive peptides generated from milk can originate both from whey proteins (β-lactoglobulin, α-lactalbumin, serum albumin, immunoglobulins, lactoferrin and protease-peptone fractions) and from caseins (α-, β- and κ-casein) [8,9,10]. Bioactive peptides are studied for their various beneficial activities, for example anti-hypertensive, anti-microbial, opioid and antioxidant [4,11,12,13,14]. The antioxidant activity of bioactive peptides can depend on their amino acid composition and position in the sequence [15]. Moreover, these compounds can exert their antioxidant activity in cell environment through activation of specific pathways [16,17]. Oxidants and electrophiles are well known molecules recognized to determine the disruption of Keap1/Nrf2 interaction [16,18,19]. However, new series of other compounds are now emerging, such as the bioactive peptides, that with specific protein-protein interactions are able to activate nuclear factor erythroid 2-related factor 2 (Nrf2). The latter, after dissociation from Kelch-like ECH-associated protein 1 (Keap1), migrates to the nucleus where interacts with the antioxidant response element (ARE), activating a large number of genes expressing antioxidant enzymes. Nrf2 translocation is one of the key events required for the regulation of Keap1/Nrf2 pathway and it is considered an evidence of the activation of the system. [20,21] 

As reported in the present paper, peptide fractions from fermented milk were isolated and tested for their antioxidant properties. From the most active fractions, sequences of the most abundant peptides were identified and then synthesized. Finally, these compounds were analyzed for their antioxidant activity in vitro and in a cellular model. 

This work aimed to understand the mechanism of action of fermented milk-derived peptides in the protection from oxidative stress. In particular, the interaction of these peptides with Keap1/Nrf2 pathway, the main molecular pathway involved in the protection of cells from oxidative stress conditions, was taken into account.

## 2. Materials and Methods 

### 2.1. Materials

DMEM + Glutamax, trypsin + EDTA (0.25%) and fetal calf serum (FBS) were purchased from Gibco (Thermo Fisher Scientific, Waltham, MA, USA). For peptide synthesis, all *N*-α-fluorenylmethyloxycarbonyl (Fmoc) L-amino acids, *p*-benzyloxybenzyl alcohol resins (Wang resin) were obtained from Merck (Darmstadt, Germany). Coupling reagent *O*-(7-azabenzotriazole-1-yl)-*N,N,N’,N’*-tetramethyluronium hexafluorophosphate (HATU) was purchased from ChemPep (Wellington, FL, USA). Formic acid was purchased from Fluka (Ammerbuch, Germany). Chemical reagents were purchased from Sigma-Aldrich (St Louis, MO, USA) and Iris Biotech (Marktredwitz, Germany). Acetonitrile, trifluoroacetic acid (TFA), 2,2′-azinobis(3-ethylbenzo-thiazoline 6-sulfonate) (ABTS), 1,1-diphenyl-2-picrylhydrazyl (DPPH), 3-(4,5-dimethylthiazolyl-2)-2,5-diphenyltetrazolium bromide (MTT), potassium persulfate, *tert*-butyl hydroperoxide (TbOOH), PBS, isopropanol, dimethyl sulfoxide (DMSO), Trolox C and Corning Incorporated Transwell^®^ 12 well plates were all obtained from Sigma-Aldrich (St Louis, MO, USA).

### 2.2. Preparation of Fermented Milk

The samples of fermented milk were collected on the day of their manufacturing. Pasteurized milk was ultrafiltered, sterilized at 120 °C for 30 s and subjected to homogenization (APV, SPX Flow Technology, Crawley, UK). At this point, milk was inoculated with 0.6 mg/L *Lactobacillus acidophilus* NCFM^®^ and 0.2 mg/L *Lactobacillus delbrueckii* subs. *bulgaricus* and *Streptococcus thermophilus* (DUPONT DANISCO, Bologna, Italy) and the product was incubated in a maturation tank at 38 °C for 10 h. At the end, fermented milk (pH 4.5) was vigorously mixed breaking the clot, and brought to 20 °C to block the fermentation process.

### 2.3. Aqueous Extract of Samples

150 mL of fermented milk were mixed with 150 mL of distilled water in an orbital shaker at room temperature (RT) for 5 min. Then the solution was centrifuged at 16,800× *g* for 25 min at 15 °C. The supernatants were filtered through Whatman Chr 1 (GE Healthcare, Chicago, IL, USA) and the obtained aqueous extracts were stored at –20 °C until use [22]. 

### 2.4. Solid Phase Extraction of Bioactive Fractions from Aqueous Preparations

For the extraction of peptide fractions, a solid phase STRATA C18 E column (Phenomenex, Torrance, CA, USA) was employed. The column was initially conditioned with 50 mL of 100% acetonitrile (ACN) and rinsed with 125 mL of 0.1% trifluoroacetic acid (TFA) aqueous solution. Aliquots of 50 mL of aqueous extract, obtained as described above, were loaded onto the column. After washing with 125 mL of 0.1% TFA, a discontinuous gradient step of ACN was applied in order to obtain peptide enriched fractions [22]. Briefly, the column was eluted with 50 mL of 5%, 30% and 50% ACN solutions and the fractions 5–30% ACN and 30–50% ACN were collected, frozen at –80 °C and lyophilized (Freeze Drier, Edwards, Burgess Hill, west Sussex, UK), then stored at –20 °C until further analysis. 

### 2.5. Purification of Peptide Fractions

In order to increase the resolution in peptide separation, the peptide enriched fractions obtained with solid phase extraction were further purified. 5–30% ACN fraction (35 mg) was dissolved in 2 mL of 0.1% TFA and further purified using preparative Reversed Phase-High Performance Liquid Chromatography (RP-HPLC) with a PrepNova-Pak^®^ HR C18 column (6 μm 60 Å 25 × 10 mm; Waters, Milford, MA, UK) with a linear gradient from 5% to 40% ACN with a flow rate of 12 mL/min. Briefly, after an isocratic step at 5% ACN for 5 min, ACN was linearly increased from 5% to 40% for 24 min and then to 100% within the following 5 min. The column effluent was monitored by UV detection (λ = 220 nm). Fractions were collected every 2 min and then lyophilized.

### 2.6. Liquid Chromatography-Tandem Mass Spectrometry (LC-MS/MS) Analysis 

The fraction of interest was dissolved in H_2_O/0.1% formic acid (FA) and an amount of sample corresponding to 1 μg (2.5 μL) was subjected to LC-MS/MS analysis. For the MS analyses a LTQ-Orbitrap XL mass spectrometer (Thermo Fisher Scientific, Waltham, MA, USA) coupled online with a nano-HPLC Ultimate 3000 (Dionex-ThermoFisher Scientific, Waltham, MA, USA) was employed. A homemade 10 cm chromatographic column packed into a pico-frit (75 μm internal diameter (I.D.), 15 μm tip, New Objective, Woburn, MA, USA) with C18 material (Aeris Peptide 3.6 μm XB-C18, Phenomenex Torrance, CA, USA) was utilized for sample loading. A linear gradient from 3% to 40% of ACN, 0.1% FA in 20 min at a flow rate of 250 nL/min was chosen for peptide elution from the original sample. Data of the LC-MS/MS analysis were obtained performing on the Orbitrap a full scan at high resolution (60,000). The MS-MS fragmentation scans were conducted on the ten most intense ions acquired with collision-induced dissociation (CID) fragmentation in the linear trap (data-dependent acquisition—DDA). Furthermore, the following parameters were set for this analysis: capillary voltage 1.2 kV; source temperature 200 °C.

### 2.7. Database Search and Peptide Identification

In order to permit peptide identification two proteomic tools, Proteome Discoverer software (version 1.4, Thermo Fisher Scientific, Waltham, MA, USA) and the Mascot Search engine server (version 2.2.4, MatrixScience, London, UK) were utilized for processing raw LC-MS/MS data files given by Xcalibur software (version 2.2 SP1, Thermo Fisher Scientific, Waltham, MA, USA). Protein identification against the SwissProt database (version 20180703; 597363 sequences; SIB Swiss Institute of Bioinformatics, Lausanne, Switzerland) was performed using the following parameters: no enzyme, precursor tolerance 10 ppm and fragment tolerance 0.6 Da. Oxidation of methionine was set as variable modification. The Percolator algorithm was used to assess the false-discovery rate and filter the results (FDR < 0.01). Proteins were grouped in protein families according to the principle of maximum parsimony. 

### 2.8. Peptide Synthesis 

All the peptides derived from alignments with proteins of bovine milk proteome were subjected to synthesis, employing a solid-phase technique performed on a fully automated peptide synthesizer (Syro II, MultiSynTech Gmbh, Witten, Germany). Wang resins preloaded with the first N-α-Fmoc-protected amino acid were utilized for stepwise assembly of the entire peptide chain. This assembly was done according to the Fmoc standard strategy and was based on the use of HATU as the coupling reagent [23,24]. The side-chain protected amino acid building blocks utilized in these peptide syntheses were Fmoc-Glu(OtBu)-OH, Fmoc-Gln(Trt)-OH, Fmoc-Asn(Trt)-OH, Fmoc-His(Trt)-OH, Fmoc-Ser(tBu)-OH, Fmoc-Lys(Boc)-OH, Fmoc-Tyr(tBu)-OH, Fmoc-Arg(Pbf)-OH, Fmoc-Asp(OtBu)-OH, Fmoc-Trp(Boc)-OH, Fmoc-Cys(Trt)-OH and Fmoc-Thr(tBU)-OH. A step of deprotection of the final peptides was conducted, followed by cleavage from the resin with a mixture of 88% (*v/v*) trifluoroacetic acid (TFA) with 5% phenol (*w/v*), 5% H_2_O (*v/v*) and 2% (*v/v*) of triisopropylsylane via shaking at RT for 2.5 h. A step of vacuum filtration allowed to remove the resin from the assembled peptide chains. Then, the peptides were precipitated with cold diethyl ether and transformed into pellet by a centrifugation procedure. Two washes with cold diethyl ether were performed on the precipitated peptides. At the end, purification of the crude peptides was done through flash chromatography (SP1, Biotage, Uppsala, Sweden) on a Biotage SNAP Ultra C18 12 g cartridge packed with Biotage HP-Sphere C18 25 μm spherical silica. A final step of molecular mass confirmation was performed by mass spectroscopy on a MALDI-TOF/TOF mass spectrometer (ABI 4800, AB Sciex, Framingham, MA, USA). The mechanism of action analysis was conducted on four selected peptides, **N-15-M**, **E-11-F**, **Q-14-R** and **A-17-E.**

### 2.9. ABTS and DPPH Scavenging 

Samples were analysed for antioxidant capacity through ABTS and DPPH tests [25]. For the first assay, 7 mM ABTS solution and 2.46 mM potassium persulfate were prepared and maintained for 18 h in the dark to obtain the radical molecule ABTS•^+^. The other test utilized a stable radical (1,1-diphenyl-2-picrylhydrazyl (DPPH)) dissolved in ethanol. Briefly, 0.1 mL of peptide solution (4 mg/mL) was added to 0.1 mL of 0.160 mM DPPH or 0.08 mM ABTS•^+^ solution. The change of absorbance was estimated at 517 nm and 415 nm for DPPH and ABTS assay, respectively, with a plate reader (Infinite^®^ M200 PRO, Tecan, Männedorf, Switzerland). For ABTS test a calibration curve was set up using Trolox C as standard and results were expressed as Trolox Equivalent Antioxidant Capacity (TEAC). In the other assay, the results were indicated as percentage of antioxidant capacity inhibition (% DPPH scavenging).

### 2.10. Caco-2 Cell Culture

Caco-2 cells were obtained from DISCOG (University of Padova, Padova, Italy). The cells cultured in DMEM (high glucose) supplemented with 10% FBS, were used between 35 and 60 passages. In order to perform transepithelial transport, 8 × 10^4^ cells were seeded on Transwell^©^ cell culture inserts (0.4 µm pore sizes, 12 mm diameter, 1.12 cm^2^ grown surface; Corning Life Sciences, Tewksbury, MA, USA). Cells were grown, differentiated for 21 days and the monolayer integrity was estimated by transepithelial electrical resistance (TEER) (Millicell^®^ ERS-2 volt-ohmmeter, EDM Millipore, Darmstadt, Germany) showing values higher than 1100 Ω × cm^2^.

### 2.11. Transepithelial Transport of Fractions or Peptides Through Caco-2 Cell Monolayers

The intestinal barrier crossing capacity of the fractions or synthetic peptides through Caco-2 cells monolayer was evaluated using Transwell^®^ insert model according to Tonolo et al. [26]. Briefly, after 21 days of culturing, Caco-2 cells differentiated forming a monolayer that delimited an upper part (apical compartment) and lower part (basolateral compartment). The monolayer was rinsed three times with Hank’s balanced salt solution (HBSS) and 10 mM D-glucose and equilibrated for 30 min at 37 °C. After, cells were incubated in the presence of 0.75 mL HBSS containing 150 µg fraction or 75 µg peptide in the apical chamber at 37 °C for 120 min. Samples collected from both compartments (apical and basolateral) at different times were centrifuged at 11,600× *g*, filtered with 0.45 µm filter, frozen and lyophilized. Subsequently, the obtained samples were dissolved in 100 µL of 0.1% TFA aqueous solution and evaluated by RP-HPLC and Matrix assisted laser desorption/ionization- time of flight/time of flight mass spectrometry (MALDI-TOF/TOF MS). 

#### RP-HPLC and MS Analyses

The apical and basolateral compartments were evaluated in RP-HPLC using a column Onyx Monolithic C18 100 mm × 4.6 mm, LC column (Phenomenex, Torrance, CA, USA) with a Waters 2695 Separation Module (Milfold, MA, USA) with a Waters 996 Photodiode Array Detector. Separations were performed with a linear gradient from 0–60% ACN in the presence of 0.1% TFA over 20 min at a constant flow rate of 2 mL/min monitoring the peaks by UV detection (λ = 220 nm).

Subsequently, the obtained fractions were analysed with a MALDI-TOF/TOF 4800 mass spectrometer (AB Sciex, Framingham, MA, USA). After an initial full MS scan, enlargements on the MS signals of interest were acquired. For the basolateral fractions, the samples, after lyophilisation, were dissolved in 50 µL of 25% ACN and 0.1% TFA. The analysis was then performed on 2 µL of these solutions mixed with 2 µL of peptide MALDI matrix α-cyano-4-hydroxycynnamic acid (10 mg/mL aqueous 70% acetonitrile/0.1% TFA). The following analytical conditions were set for MALDI-TOF spectra acquisition: positive ion reflector mode, initial mass range 500–3500 Da (the mass range was then adapted for each enlargement), variable laser intensity (3000–3800), shots/sub-spectrum 50, total shots/spectrum 1500 and accelerating voltage of 20 kV. Final data analysis and fragments identification were done on Data Explorer software (AB Sciex, Framingham, MA, USA), utilizing external mass calibration performed with mass peptide standards (Sigma-Aldrich, St Louis, MO, USA). 

### 2.12. Cell Viability 

The analysed peptide fractions and synthetic peptides were tested with MTT assay. Caco-2 cells (1 × 10^4^) were seeded in 96 well plates and incubated with peptide fractions (0.125 mg/mL) or synthetic peptides (0.05 mg/mL). After 24 h, medium was removed and MTT solution (0.5 mg/mL, 100 μL) in PBS (1×) was added for 3 h in the dark at 37 °C. Removed MTT solution, the reaction was stopped with 100 µL of isopropanol/DMSO (9:1). The absorbance was followed (Abs_595–690_) using a plate reader (Tecan Infinite^®^ M200 PRO, Männedorf, Switzerland). 

### 2.13. ROS Production Estimation

ROS production in Caco-2 cell line was measured by using 5-(and 6)-chloromethyl-20,70-dichlorohydrofluorescein diacetate (CM-H_2_DCFDA,Molecular Probes, Thermo Fisher Scientific, Waltham, MA, USA). Briefly, cells (1 × 10^4^) were grown in a 96-wells plate for 48 h, and then treated with synthetic peptides (0.05 mg/mL) for 24 h. Cells washed in PBS 1×/10 mM glucose were loaded with 10 µM CM-H_2_DCFDA for 20 min in the dark at 37 °C. Subsequently, the fluorescent probe was removed and cells were rinsed with PBS 1×/10 mM glucose and subjected to oxidative stress in the presence of 250 µM TbOOH. Fluorescence increase was followed at 485 nm (λ excitation) and 527 nm (λ emission) for 90 min using a plate reader (Tecan Infinite^®^ M200 PRO, Männedorf, Switzerland).

### 2.14. Nrf2 Translocation to the Nucleus

In order to investigate the Keap1/Nrf2 activation, the translocation of Nrf2 to the nucleus was followed. For this purpose, nuclear and cytosolic fractions were divided according to the method described by Yao et al. (2014), with some modifications [27,28]. Briefly, Caco-2 cells (1 × 10^6^) were grown in T25 flasks for 48 h and then treated with 0.05 mg/mL **N-15-M**, **E-11-F**, **Q-14-R** and **A-17-E.** After 24 h, cells were rinsed with 1 mL of PBS 1× and lysed for 15 min on ice with 100 µL of buffer containing 10 mM Hepes/Tris pH 7.9, 0.1 mM EGTA, 0.1 mM EDTA, 0.1 mM PMSF, 10 mM KCl, 1 mM NaF and a protease inhibitor cocktail (Complete, Roche^®^, Basel, Switzerland). The samples were rapidly added of IGEPAL (5% final concentration), mixed for 15 s and centrifuged at 1000× *g* for 10 min at 4 °C. The pellet (nuclear fraction) was dissolved in 20 mM Hepes/Tris (pH 7.9), 1 mM EGTA, 1 mM EDTA, 0.4 M NaCl in the presence of 0.1 mM PMSF, 1 mM NaF and protease inhibitors (Complete, Roche^®^, Basel, Switzerland). Samples were mixed every 2 min for 10–15 s and centrifuged at 20,000× *g* for 10 min at 4 °C to discard the debris. Nuclear proteins (30 µg evaluated according to Lowry et al. [29]) were subjected to SDS-PAGE (10%) and subsequently to western blot analysis to define the expression level of Nrf2. Densitometric analysis of WB was carried out using NineAlliance software (Mini 9 17.01 version, Uvitec Alliance, Cambridge, UK). PCNA was used as loading reference.

### 2.15. Cell Lysates

Cells (5 × 10^5^ per well) were seeded in six well plates and, after 48 h, treated with 0.05 mg/mL **N-15-M**, **E-11-F**, **Q-14-R** and **A-17-E**. After 24 h, cells were collected, rinsed with 1 mL of PBS 1× and then lysed with modified RIPA buffer in the presence of protease inhibitor cocktail (Complete, Roche^®^, Basel, Switzerland) [19]. Protein content was estimated with the Lowry method [29]. 

#### 2.15.1. Antioxidant Enzymes Detection

Superoxide dismutase (SOD1), thioredoxin reductase 1 (TrxR1), glutathione reductase (GR) and NAD(P)H quinone dehydrogenase 1 (NQO1) were evaluated by Western blot analysis, using 30 µg of cell protein lysates subjected to SDS-PAGE (12%). The WB detection was assessed using ECL system with UVITEC (Alliance Q9 Advanced, Cambridge, UK) equipment. Densitometric quantification of antioxidant enzymes WB was performed using NineAlliance software (Mini 9 17.01 version, Uvitec Alliance, Cambridge, UK). GAPDH was used as loading reference.

#### 2.15.2. TrxR1 and GR Activities

TrxR1 and GR activities were measured spectrophotometrically using 50 µg of cell lysates proteins and following absorbance at 412 and 340 nm, respectively as described by Tonolo F. 2018 [26]. 

### 2.16. Gene Expression Analysis

The levels of gene expression of antioxidant enzymes (TrxR1, GR, NQO1 and SOD1) were evaluated with Real-Time PCR and β-actin was used as reference. Firstly, Caco-2 cells (5 × 10^5^) were cultured into six well plates for 48 h in a complete medium and then treated with **N-15-M**, **E-11-F**, **Q-14-R** and **A-17-E** (0.05 mg/mL) for 24 h. Cells were rinsed with 1 mL PBS 1×, lysed with 1 mL of TRIzol reagent (Invitrogen, Thermo Fisher Scientific, Waltham, MA, USA) and transferred into a new test tube. At this point, 0.2 mL of chloroform were added and vigorously mixed for 15 s. Then samples were placed at RT for 15 min and subsequently centrifuged at 12,000× *g* for 15 min at 4 °C. In this way, three phases were obtained and the upper one, that contain mRNA, was transferred in a new test tube. Samples were added of 0.5 mL of isopropanol, mixed and maintained at RT for 10 min and subsequently centrifuged at 12,000× *g* for 10 min at 4 °C. The pellets were rinsed with 1 mL of 75% ethanol and mixed vigorously for 1 min. Samples were centrifuged at 7500× *g* for 10 min at 4 °C and the pellets were air-dried. The extracted mRNA was diluted in 0.01 mL of RNase free water and its concentration was estimated using NanoDrop system (Thermo Fisher Scientific). mRNA (1 µg) was subjected to reverse transcription using Euroscript M-MLV Reverse Transcriptase in the presence of 25 µg/mL oligo(dT), 10 mM DNTp mix, 10 mM DTT and RNase inhibitors (Euroclone, Milan, Italy) in a final volume of 0.02 mL. Before adding the reverse transcriptase, the mRNA was denatured at 65 °C for 5 min, then the mix were placed at 42 °C for 50 min and at 70 °C for 15 min. The resulting cDNA (1.5 ng/µL) was used for the Real-Time PCR analysis using Hot FIREpol Eva Green qPCR Supermix (Solis BioDyne, Tartu, Estonia). The target cDNA was amplified as follow: an initial step for polymerase activation at 95 °C for 12 min and then 40 cycles of denaturation for 15 s at 95 °C, annealing at 65 °C for 1 min and elongation at 72 °C for 1 min. All the primers were purchased from Sigma-Aldrich (St Louis, MO, USA). GR: Fw: 5′-TCA CGC AGT TAC CAA AAG GAA A-3′, Rv: 5′-CAC ACC CAA GTC CCC TGC ATA T-3′; TrxR1: Fw: 5′-GCC CTG CAA GAC TCT CGA AAT TA-3′, Rv: 5′-GCC CAT AAG CAT TCT CAT AGA CGA-3′; NQO1: Fw: 5′-GGA GAC AGC CTC TTA CTT GCC AAG, Rv: 5′-CCA GCC GTC AGC TAT TGT GGA TAC; β-actin: Fw: 5′-ACC TGA CTG ACT ACC TCA TGA AGA-3′, Rv: 5′-GCG ACG TAG CAC AGC TTC TC-3′; SOD 1: Fw: 5′-TCA GGA GAC CAT TGC ATC ATT-3′, Rv: 5′-CGC TTT CCT GTC TTT GTA CTT TCT TC-3′.

### 2.17. Molecular Docking Analysis

The interaction of peptides **N-15-M**, **E-11-F**, **Q-14-R** and **A-17-E** with the Kelch domain of Keap1 was investigated trough docking simulation. The protein-peptide model was initially predicted using CASB-Dock [30], providing the peptide sequence and the chain X of the Crystal structure of Kelch domain of Keap1 bound to Neh2 domain of Nrf2 (2FLU), and refined with HADDOCK [31,32]. After 50 ns of relaxation with gromacs 2016.1 [33], the interactions between peptides and their target were evaluated using PISA [34] and UCSF Chimera [35].

### 2.18. Statistical Analysis

All the reported results are indicated as the mean values ± SD of at least three independent experiments. The analysis of variance was estimated by multiple comparison test employing Tukey-Kramer test and the differences with *p* < 0.05 were considered significant. The software InStat 3 (GraphPad Software, San Diego, CA, USA) was used.

## 3. Results

Aqueous extracts from fermented milk, obtained with three different microbial strains *Lactobacillus acidophilus*, *Lactobacillus delbrueckii subs. bulgaricus* and *Streptococcus thermophilus,* were used. Then, the samples underwent two different types of purification. To obtain peptide enriched fractions, the first one was a discontinuous step gradient of ACN (5–30% and 30–50%). The major amount of peptides was found in the 5–30% ACN step, while in the 30–50% fraction only a small amount of peptides was present. Subsequently, to provide a higher resolution in peptide separation, the fractions were further purified with RP-HPLC using a continuous gradient. The collected samples were tested for their antioxidant properties in vitro and in Caco-2 cells. The peptides present in the most active fraction were identified.

### 3.1. Analysis of 5–30% ACN Fraction in Caco-2 Cells: Evaluation of Cell Viability

To estimate the effects of 5–30% and 30–50% ACN fractions on Caco-2 cells, the MTT test was performed. Caco-2 cells (1 × 10^4^) were treated with the peptide fractions (0.125 mg/mL) and, as shown in Table 1, both fractions were not cytotoxic. Moreover, when cells (1 × 10^4^) were subjected to oxidative stress induced by 200 µM TbOOH we observed a decrease of viability. However, when cells were pretreated with the 5–30% ACN fraction a significant protective effect from oxidative stress was observed. Therefore, the further analysis was conducted only on this fraction.

### 3.2. HPLC Analysis, Antioxidant Properties In Vitro and In a Cellular Model of the Purified Fractions Obtained From the 5–30% ACN Pool

**Figure 1:**
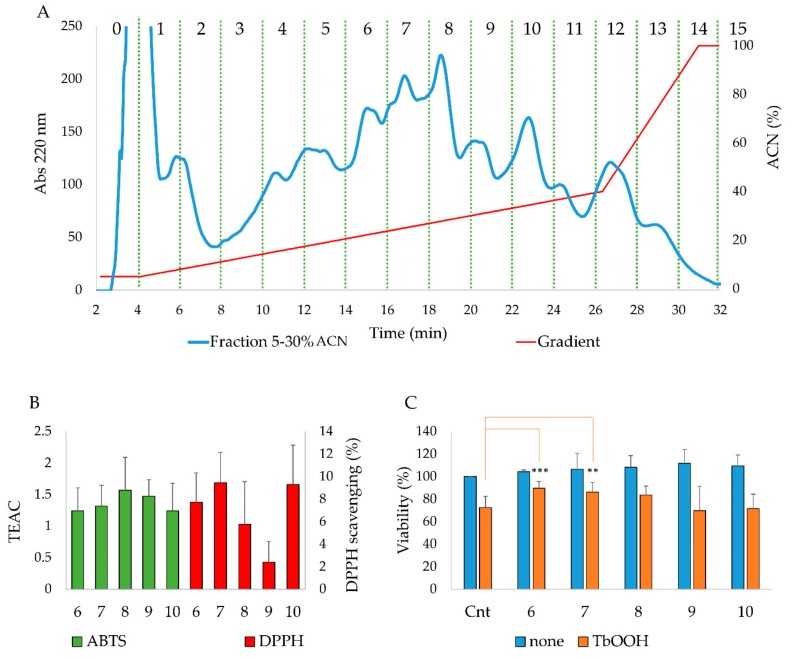
**(A**)Purification of the 5–30% ACN fraction with RP-HPLC. Fractions were collected every 2 min. (**B**) Analysis of antioxidant capacity of the purified fractions in vitro with 2,2′-azinobis(3-ethylbenzo-thiazoline 6-sulfonate) (ABTS) (green) and 1,1-diphenyl-2-picrylhydrazyl (DPPH) (red) scavenging tests. (**C**) Effects of the purified fractions on cell viability in the presence and absence of TbOOH. Caco-2 cells were treated with the indicated fractions for 24 h and oxidative stress was induced by 200 µM TbOOH. Means of at least three experiments (eight replicates for each experiment) were compared with the treated control. (*** *p* < 0.001, ** *p* < 0.01).

Fraction 5–30% ACN, containing a large amount of peptides, was further purified, in order to isolate and identify the most active peptides. To this purpose, 35 µg of the 5–30% ACN fraction was subjected to RP-HPLC (PrepNova-Pak^®^ HR C18) employing a linear gradient from 5% to 40% ACN with a flow rate of 12 mL/min. Collecting the eluted solution every two minutes, fifteen fractions were obtained as described in Figure 1A. In particular, fractions from 0 to 5 and from 11 to 15 were discarded, because the amount of obtained peptides was negligible and insufficient to perform further experiments, indicating a low peptide content at the beginning and at the end of the gradient. On the other hand, each fraction from 6 to 10 was analyzed for its antioxidant properties in vitro. As reported in Figure 1B, all the fractions showed an antioxidant capacity in vitro, as they exhibited moderate TEAC and DPPH scavenging values. Moreover, the effects on Caco-2 cells of the purified fractions were analyzed and the peptides did not show cytotoxicity (Figure 1C). In addition, all the fractions were evaluated for their protection from oxidative stress in cells pretreated with them and subsequently incubated with 200 µM TbOOH. As shown in Figure 1C, some fractions, in particular 6, rescued the viability of Caco-2 cells treated with the oxidative agent. For this reason, this fraction was selected for the further analysis. In order to identify the peptides, present in the most active fraction (6) and able to cross the intestinal barrier, the Transwell^®^ insert model was used. Caco-2 cells were grown on the Transwell^®^ insert for 21 days to reach the differentiated epithelium formation and peptide fractions were added in the apical compartment. After 10 and 120 min, apical and basolateral solutions were collected and analyzed by HPLC and mass spectrometry. As shown in Figure S1, some peptides present in the fraction 6 can cross Caco-2 monolayer.

### 3.3. Identification of the Peptides with Mass Spectrometry Analysis 

Then, peptides included in fraction **6** were analyzed with mass spectrometry in order to identify their sequence. The investigation with the Proteome Discoverer and Mascot gave a list of peptides reported in the Table S1. The list of peptides for each protein given by the proteomic identification was aligned with the sequences of the reference proteins (Figure S2) and the candidates for the solid-phase peptide synthesis were chosen in order to obtain the maximum protein sequences coverage. Some criteria were considered as fundamental for the choice of candidate peptides, such as the maximum protein sequences coverage, the best match score between the peptides given by the Proteome Discoverer Software and the reference proteins expressed by Peptide Spectral Match (#PSM), and the peptide length. When more than one peptide covered a region, only the peptide that has the best match score, expressed by the highest value of #PSM was chosen. Of note, the same analysis were performed for fraction 7, but the identified peptides were mostly the same as those identified in fraction 6. Due to the more significant activity in cellular model of fraction 6, the further experimentation was performed with the latter. The sequences and properties of the synthesized peptides were reported in Table 2. 

### 3.4. New Identified Peptides

From the reported analysis, 23 peptides were chosen and synthetized. Of them, 15 were not retrieved on the BIOPEP database, indicating that they were completely new in the scientific investigation. The remaining 8 were already registered in the sequence database, although with activities different from the antioxidant one. These peptides were tested for their antioxidant properties in vitro and in the Caco-2 cells model. In particular, 4 of them were selected for their antioxidant effects exerted on the cells and further analyzed to understand their mechanism of action.

#### 3.4.1. Antioxidant Properties In Vitro and In Caco-2 Cells

The 23 peptides were evaluated for their antioxidant capacity using ABTS and DPPH scavenging tests. As reported in Table 3, many peptides displayed a great antioxidant capacity with different extent, while some of them were ineffective. All the synthetic peptides were also tested in the cellular model for their potential cytotoxicity. In addition, they were also checked for their capability to protect against oxidative stress induced by 200 µM TbOOH. As reported in Table 3, column c, all peptides were not cytotoxic. Moreover, most of the synthetized peptides protected the viability of the cells once treated with the oxidative agent, in particular **V-12-Q**, **N-15-M**, **E-11-F**, **K-15-L** and **I-14-L**. These peptides showed a recovery of viability of about 10% (Table 3, column d).

#### 3.4.2. Inhibition of ROS Production by Bioactive Peptides

**Figure 2:**
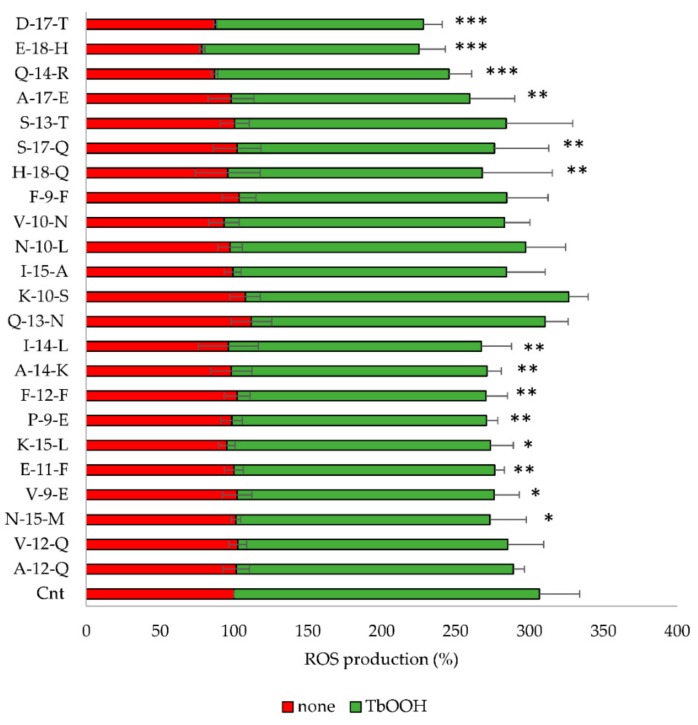
Estimation of reactive oxygen species (ROS) production in Caco-2 cells treated with the indicated peptides (0.05 mg/mL) in the absence (red) or presence (green) of 250 µM TbOOH. The values at 5000 s were reported and the means of at least three experiments (four replicates for each experiment) were compared with the treated control. (*** *p* < 0.001, ** *p* < 0.01, * *p* < 0.05).

The identified peptides were further examined for their capacity to inhibit ROS production in Caco-2 cells. To this purpose, Caco-2 cells were treated with the peptides for 24 h and subsequently incubated with CM-DCFDA as described in the Material and methods. ROS production in Caco-2 cells pretreated with the peptides was similar to the untreated control, while, when the oxidative stress was induced by 250 µM TbOOH we observed a marked decrease in fluorescence after pretreatment with **N-15-M**, **E-11-F**, **Q-14-R**, **E-18-H**, **H-18-Q**, **A-17-E**, **D-17-T**, **S-17-Q**, **V-9-E**, **P-9-E** and **F-12-F** (Figure 2). 

### 3.5. Analysis of the Mechanism of Action of the Antioxidant Peptides

Considering both the protection of the viability and the inhibition of ROS production in Caco-2 cells, **N-15-M**, **E-11-F**, **Q-14-R** and **A-17-E** were selected for their powerful antioxidant effects. Therefore, these four peptides were further analyzed to define their mechanism of action.

#### 3.5.1. Nrf2 Translocation to the Nucleus

**Figure 3:**
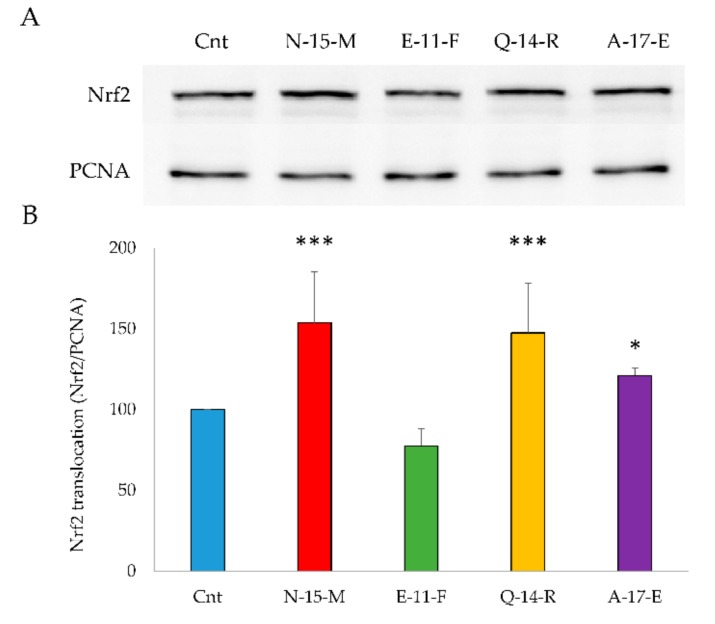
Nrf2 translocation from cytosol to nucleus in Caco-2 cells in the presence of **N-15-M**, **E-11-F**, **Q-14-R** and **A-17-E**. (**A**) Cells were treated with 0.05 mg/mL of each peptide for 24 h. Nuclear fractions were isolated and proteins were subjected to WB detection as indicated in paragraph 2.15. (**B**) Densitometric analysis of four experiments compared with the control were reported, using PCNA as loading control. (*** *p* < 0.001, * *p* < 0.05).

An antioxidant action inside the cell could be due to activation of the Keap1/Nrf2 pathway. For this reason, the translocation of Nrf2 from the cytosol to the nucleus in Caco-2 cells treated with the four peptides **N-15-M**, **E-11-F**, **Q-14-R** and **A-17-E** (0.05 mg/mL) was evaluated. Cells (1 × 10^6^) were treated for 24 h with the peptides and then processed to obtain the nuclear fraction, as described in Materials and methods. Nrf2 present in the nuclear fraction was detected by Western blot analysis. Peptides **N-15-M**, **Q-14-R** and **A-17-E** increased significantly the levels of Nrf2 in the nucleus as reported in Figure 3, while **E-11-F** was completely ineffective.

#### 3.5.2. Antioxidant Enzymes Gene Expression Analysis

After the observation that **N-15-M**, **Q-14-R** and **A-17-E** showed a large increase of Nrf2 in the nucleus, the levels of gene expression of antioxidant enzymes were analyzed by RT-PCR. Cells (5 × 10^5^) were treated for 24 h with the four peptides and processed as described in Section 2.16. The transcription of these enzymes is regulated by the translocation of Nrf2 to the nucleus where it can bind ARE. 

**Figure 4:**
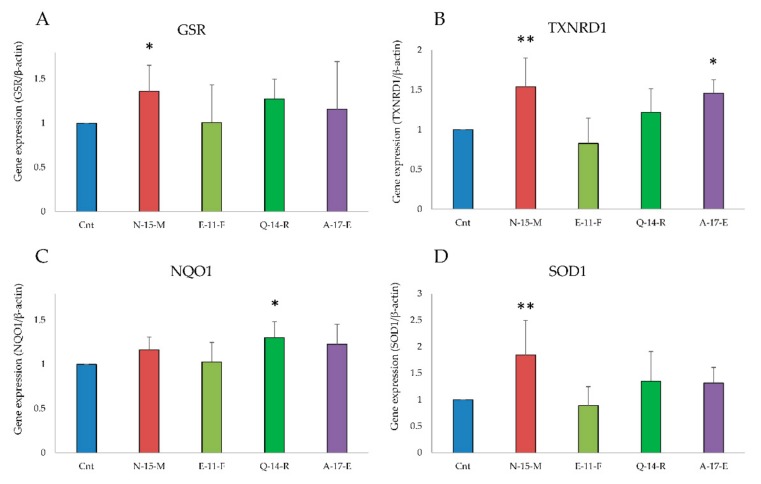
Antioxidant enzymes gene expression analysis. The gene expression of glutathione reductase (GRS, **A**), thioredoxin reductase (TXNRD1, **B**), NADPH quinone oxidoreductase (NQO1, **C**) and superoxide dismutase (SOD1, **D**) was evaluated in cDNA obtained from Caco-2 cells treated with **N-15-M**, **E-11-F**, **Q-14-R** and **A-17-E** (0.05 mg/mL) for 24 h. β-actin was used as reference. Means of at least four experiments were compared with the control. (** *p* < 0.01, * *p* < 0.05).

As shown in Figure 4, the peptides **N-15-M**, **Q-14-R** and **A-17-E** were able to induce an increase of the *GSR, TXNRD1, NQO1* and *SOD1* mRNA levels. On the other hand, **E-11-F**, that did not show any effect on the translocation of Nrf2 to the nucleus, also in this case did not exert any effect on the antioxidant enzymes gene expression.

#### 3.5.3. Antioxidant Enzymes Detection in the Presence of the Four Peptides

**Figure 5:**
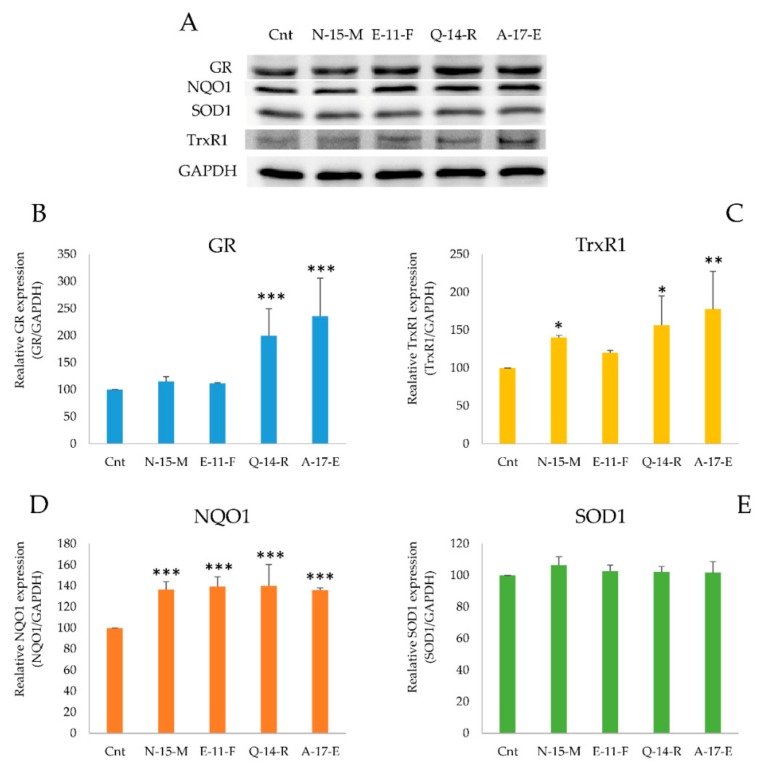
Antioxidant enzymes detection by WB analysis. (**A**) Protein levels of glutathione reductase (GR), thioredoxin reductase (TrxR1), NADPH quinone oxidoreductase (NQO1) and superoxide dismutase (SOD1) in Caco-2 cell lysates treated with the four peptides (0.05 mg/mL) for 24 h. (**B–E**) Densitometric analysis of four experiments were compared with the control and normalized using GAPDH as loading control. (*** *p* < 0.001, ** *p* < 0.01, * *p* < 0.05).

In order to confirm the observation regarding the increase of gene expression induced by **N-15-M**, **Q-14-R** and **A-17-E**, western blot analysis of lysates of cells treated with the four peptides was performed. Cells (5 × 10^5^) were incubated in the presence of the four peptides (0.05 mg/mL) for 24 h. Aliquots of the samples (30 µg) were subjected to WB to detect glutathione reductase (GR), NADPH quinone oxidoreductase (NQO1), superoxide dismutase (SOD1) and thioredoxin reductase 1 (TrxR1). As shown in Figure 5, the most active peptides enhanced also the protein levels of these enzymes. In particular, cells treated with **Q-14-R** and **A-17-E** showed a large increase of GR, TrxR1 and NQO1 protein levels (Figure 5). 

#### 3.5.4. TrxR1 and GR Activities in Cell Lysates

**Figure 6:**
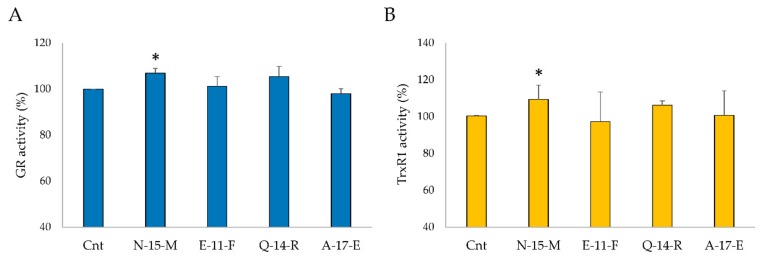
GR (**A**) and TrxR1 (**B**) activities in Caco-2 cells treated with **N-15-M**, **E-11-F**, **Q-14-R** and **A-17-E** (0.05 mg/mL). The activity of the two antioxidant enzymes was analyzed in Caco-2 cells treated with the four peptides for 24 h. Means of at least four experiments were compared with the control. (* *p* < 0.05).

The activities of TrxR1 and GR in cells treated with **N-15-M**, **E-11-F**, **Q-14-R** and **A-17-E** were also analyzed. Cells were incubated with the indicated peptides in the same conditions as described above and 50 µg of protein cell lysates were used to determine TrxR1 activity by following DTNB reduction at 412 nm and NADPH oxidation at 340 nm for GR. As showed in Figure 6, a slight increase of the activities of the two antioxidant enzymes was observed in cells treated with **N-15-M** and **Q-14-R.**

#### 3.5.5. Molecular Docking Analysis

**Figure 7:**
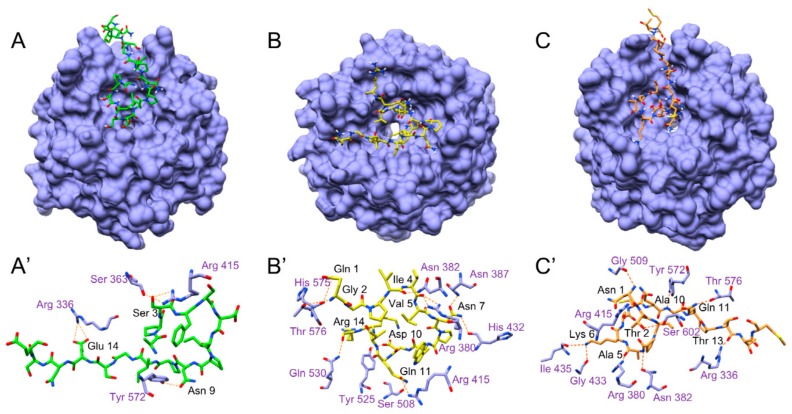
Molecular docking analysis of the interaction between peptides and Keap1 Kelch domain. (**A–C**) Binding geometry of **A-17-E**, **Q-14-R** and **N-15-M** in the pocket of Keap1. (**A’–C’**) Magnification of the interaction of Keap1 Kelch domain with **A-17-E** (**A’**), **Q-14-R** (**B’**) and **N-15-M** (**C’**). Amino acids involved in the hydrogen bond formation were connected with orange dashed lines and highlighted in Table 4.

In order to get a prediction of the interaction of the Kelch domain of Keap1 with **N-15-M**, **E-11-F**, **Q-14-R** and **A-17-E,** docking simulation was performed (Figure 7). The free energy of dissociation of the assemblies calculated after a simulation of 50 ns showed that peptide **E-11-F** has no binding affinity with the target protein. On the contrary, peptides **A-17-E**, **Q-14-R** and **N-15-M** formed stable assemblies with the Kelch domain of Keap1. More in details, the first Serine (Ser 3) of **A-17-E** formed hydrogen bonds with a serine (Ser 363) and an arginine (Arg 415), the first **A-17-E** asparagine (Asn 9) formed a hydrogen bond with a tyrosine (Tyr 572) and the inner glutamic acid (Glu 14) formed hydrogen bonds with another arginine (Arg 336) (Figure 7A’). The **Q-14-R** peptide formed hydrogen bonds between its backbone and asparagine (Asn 382), arginine (Arg 380), glutamine (Gln 530), threonine (Thr 576) and histidine (His 575) of the Keap1 pocket; there were also hydrogen bonds between the asparagine (Asn 7) of the peptide and asparagine (Asn 387), arginine (Arg 380) and histidine (His 432) of Kelch domain, the aspartic acid (Asp 10) formed a hydrogen bond with a tyrosine (Tyr 525) and the inner glutamine of the peptide (Gln 11) formed hydrogen bonds with an arginine (Arg 415) and a serine (Ser 508) (Figure 7B’). The backbone of the **N-15-M** formed hydrogen bonds with an asparagine (Asn 382) a glycine (Gly 509), a tyrosine (Tyr 572) and three arginines (Arg 336, Arg 380, Arg 415); the first threonine of the peptide (Thr 2) formed a hydrogen bond with a Serine (Ser 602), the lysine (Lys 6) of the peptide form hydrogen bonds with a glycine (Gly 433) and an isoleucine (Ile 435), the glutamine of the peptide (Gln 11) formed a hydrogen bond with a threonine (Thr 576) (Figure 7C’). It is crucial to notice that there is a certain recurrence among the residues of the Kelch domain involved in the interaction with the peptides. Moreover, these residues are also implicated in the interaction between Kelch domain and Nrf2 [37] (Table 4).

#### 3.5.6. Peptide Absorption Analysis

**Figure 8:**
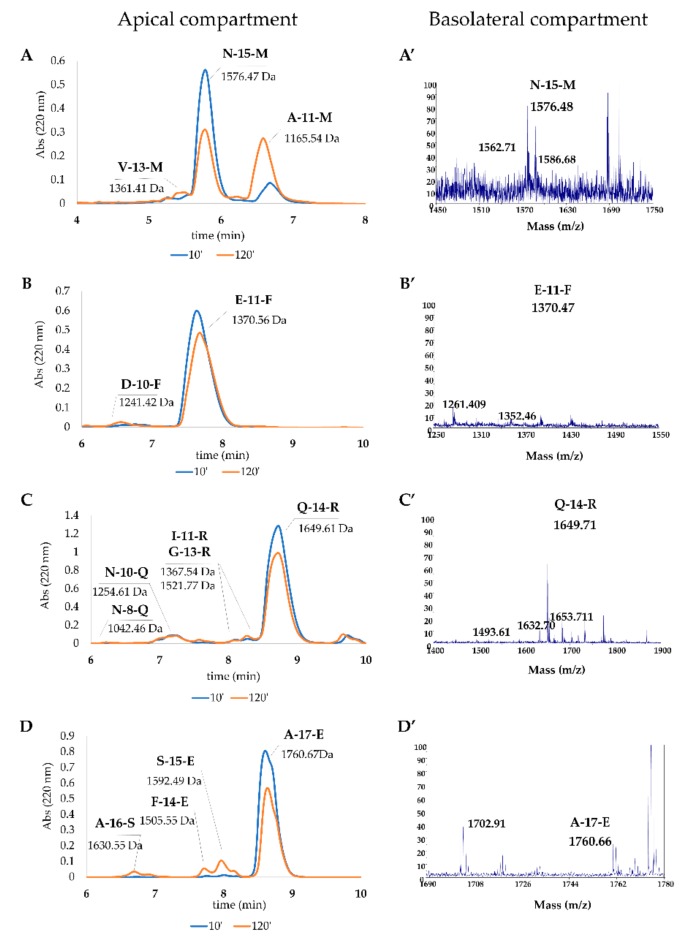
Uptake of the peptides by Caco-2 cells monolayer and their detection in AP and BL compartments. Each peptide (75 µg) was administered to the monolayer cells and samples of AP and BL were collected at the indicated time. (**A–D**) RP-HPLC chromatograms of the four peptides in the apical compartment at 10 and 120 min. (**A’–D’**) MS analysis of the peptides present in the basolateral side after 120 min.

In order to evaluate the intestinal crossing capacity of the four peptides, Transwell^®^ insert model peptide absorption, was performed. For this purpose **N-15-M**, **E-11-F**, **Q-14-R** and **A-17-E** (75 µg) were added to the differentiated Caco-2 cells grown (21 days) on the Transwell^®^ insert. After 10 min and 120 min, apical and basolateral compartments were collected and analyzed by RP-HPLC and mass spectrometry. As shown in Figure 8, all peptides, although with different extent and fragmentation pattern, can cross the intestinal barrier model. 

In the apical compartment (AP), the measured amount of **N-15-M**, **E-11-F**, **Q-14-R** and **A-17-E** after 120 min was 60.74%, 88.28%, 87.59% and 68.4%, respectively of the total amount of peptide added (Table 5). These values were due both to the absorption and to a slight fragmentation depending on the action of the brush border peptidases present in the AP. In particular, **N-15-M** gave rise to **V-13-M** and **A-11-M** and **Q-14-R** originated **N-8-R**, **V-10-R**, **I-11-R** and **G-13-R,** and, finally, **A-16-S**, **S-15-S** and **F-14-E** were produced from **A-17-E**. The peptide **E-11-F** gave rise to one fragment, **D-10-F** detectable only by mass spectrometry analysis. The increasing amount of **V-13-M** and **A-11-M** (**N-15-M)** and **A-16-S**, **S-15-E** and **F-14-E** (**A-17-E**) was visible at 120 min (Figure 8A,D, respectively). On the other hand, after 120 min a great amount of the four peptides was found in the basolateral compartment both with HPLC and with mass spectrometry analysis (Figure 8). In particular, **N-15-M**, **E-11-F**, **Q-14-R** and **A-17-E** were estimated to be 0.13%, 0.21%, 0.02% and 0.05% of the initial amount of the peptides, added in the apical compartment (Table 5). All the details about peptides and their fragments absorption analysis were reported in Table 5.

## 4. Discussion

In order to identify new bioactive peptides from fermented milk, antioxidant peptide enriched fractions were extracted and further purified with the aim of identify the sequence of the included peptides. More in detail, the fraction 5–30% ACN was selected for the high presence of peptides able to prevent oxidative stress. In fact, when Caco-2 cells were pretreated for 24 h with enriched peptide fractions, a protective effect on the viability was apparent when oxidative stress was induced by TbOOH (Table 1). Therefore, 5–30% ACN fraction was further purified by RP-HPLC obtaining five major fractions, selected on the basis of the highest peptide relative abundance (Figure 1). These collected fractions, called **6**, **7**, **8**, **9** and **10**, were studied for their antioxidant properties in vitro and in Caco-2 cells (Figure 1). In particular, fraction **6** showed a powerful activity in protecting cells from oxidative stress and thus this fraction was selected for the following studies. The sequences of the peptides included in fraction **6** were identified by using LC-MS/MS analysis and the appropriate software. From these data, 23 peptides were chosen according to the peptide coverage for a specific sequence of the reference proteins (see Supplementary Materials). The novelty of the peptides was demonstrated by their absence in the BIOPEP database. When present, their function was not related to an antioxidant effect. Three criteria have been considered for peptide selection: firstly, the maximum protein sequences coverage, in order to map each protein sequence as much as possible from N-terminus to C-terminus; secondly, the best match score, indicated by #PSM, between the peptides given by the Proteome Discoverer Software and the reference proteins; thirdly, the length of peptides, as peptides with less than 20 residues were preferred instead of longer ones. Peptide sequences derived from alignments with other proteins, such as short isoform of polymeric immunoglobulin receptor, glycosylation-dependent cell adhesion molecule, histatherin and 3-phosphoshikimate 1-carboxyvinyltransferase were not taken into account, as they were considered less important. Subsequently, the selected peptides were synthesised, characterized and tested for their antioxidant properties. Two in vitro antioxidant tests, ABTS and DPPH, were performed and, as apparent in Table 3, peptides **Q-13-N**, **K-10-S**, **I-15-A**, **N-10-L**, **F-9-F**, **Q-14-R**, **N-15-M**, **F-12-F** and **I-14-L** were the most active in the scavenging tests (in vitro). However, when all the 23 peptides were screened in a cellular model for their action against oxidative stress induced by TbOOH testing cell viability (Table 3), only peptides **V-10-N**, **Q-14-R**, **A-17-E**, **A-12-Q**, **V-12-Q**, **N-15-M**, **V-9-E**, **E-11-F** and **K-15-L** were effective. Subsequently, in order to confirm the antioxidant power of the peptides, ROS production was evaluated in cells pre-treated for 24 h with the peptides and then subjected to an oxidative stimulus induced by TbOOH. As shown in Figure 2, the most active peptides against ROS production were **N-15-M**, **E-11-F**, **Q-14-R**, **E-18-H**, **H-18-Q**, **A-17-E**, **D-17-T**, **S-17-Q**, **V-9-E**, **P-9-E** and **F-12-F**. From the analysis of all the obtained results (in vitro and in cell environment), **N-15-M**, **E-11-F**, **Q-14-R** and **A-17-E** emerged as the most active peptides. For this reason, these four peptides were used for further analysis. In particular, the mechanism of action of the selected bioactive peptides, involved in the protective effects against oxidative stress in Caco-2 cells, was investigated. We focused on Keap1/Nrf2 pathway because it is the main regulatory system in oxidative stress conditions. In fact, when an oxidative imbalance occur, Keap1 and Nrf2 dissociate and Nrf2 translocates to the nucleus where it can bind ARE, promoting the overexpression of antioxidant enzymes such as glutathione reductase (GR), NADPH quinone oxidoreductase (NQO1), superoxide dismutase (SOD1) and thioredoxin reductase 1 (TrxR1). Therefore, to understand the involvement of Keap1/Nrf2 pathway, the translocation of Nrf2 from the cytosol to the nucleus was considered in cells treated with **N-15-M**, **E-11-F**, **Q-14-R** and **A-17-E** for 24 h. As result, **N-15-M**, **Q-14-R** and **A-17-E** increased the levels of Nrf2 present in the nucleus (Figure 3) suggesting that they activate the Keap1/Nrf2 system. Subsequently, as the amount of Nrf2 in the nucleus increased, we observed also an increase of antioxidant enzymes gene expression. To this purpose, GR, TrxR1, NQO1 and SOD1 gene expression was measured in Caco-2 cells treated with the four peptides for 24 h and again **N-15-M**, **Q-14-R** and **A-17-E** were able to increase the gene expression levels of the antioxidant enzymes (Figure 4) and the consequent protein expression (Figure 5) estimated with WB analysis. Moreover, TrxR1 and GR enzymatic activities were measured in cells treated in the same conditions and **N-15-M** and **Q-14-R** increased the activity of the tested antioxidant enzymes (Figure 6). In order to confirm our observations, molecular docking analysis between the structure of Keap1 and the four peptides was performed (Figure 7). The results showed that **N-15-M**, **Q-14-R** and **A-17-E**, but not **E-11-F**, interacted with Keap1 in the Kelch domain with specific amino acid residues, involved also in the binding between Keap1 and Nrf2 (Table 4). The Kelch repeats sequence of Keap1 (AA 327–609) is responsible for the binding to Nrf2 which participates with the DLG (AA 29–31) and ETGE (AA 79–82) motifs [21]. In particular, the ETGE motif guarantees a strong binding of Nrf2 to Keap1. Specific amino acids residues in the Kelch repeats (especially Arg380 and Arg415) facilitate the binding to the transcription factor. Our peptides are able to interact with Keap1 sequence with many of the amino acids involved in the binding to the ETGE motif. As apparent, **Q-14-R** interacts with Arg 380, Asn 382, Arg 415, Ser 508, Tyr 525 and Gln 530, while peptide **N-15-M** interacts with Arg 380, Asn 382, Arg 415, Tyr 525 and Ser 602. Furthermore, the bioactive peptides examined are able to interact with several other amino acids of the Kelch domain such as Arg 336, Asn 387, His 432, Gly 433, Ile 435, Gly 509, His 575 and Thr 576. Of note, molecular docking approach showed that **E-11-F** did not interact with Keap1. 

The overall results suggested that the antioxidant effects highlighted in the cells treated with **N-15-M**, **Q-14-R** and **A-17-E** were due to the interaction of the bioactive peptides with the Keap1 pocket, which causes the disruption of the binding with Nrf2 and the subsequent activation of the signaling cascade. All these findings were in agreement with our previous results [28].

Finally, we studied the capability of these peptides to cross the intestinal barrier. Using the Transwell^®^ technique, we administered the four peptides to differentiated Caco-2 cells in the apical compartment (Figure 8). After 10 and 120 min the apical and basolateral compartments were collected and analyzed by RP-HPLC and mass spectrometry. We observed that an appreciable amount of peptides were able to reach the basolateral compartment partly with modifications as showed by the mass spectrometry analysis (Figure 8). Table 5 reports in detail each peptide and breakdown fragments. More specifically, **N-15-M** and **E-11-F** showed the best intestinal barrier crossing capacity. In fact, the 0.13% and 0.21% of these peptides reached the basolateral compartment, respectively. On the other hand, **Q-14-R** and **A-17-E** underwent a slight fragmentation by the brush border peptidases and only the 0.02% and 0.05%, respectively, reached the basolateral compartment. This observation leads to think that the peptides ingested orally can reach the blood circulation and, if not cleaved, they can exert their beneficial effects in many organs and tissues.

## 5. Conclusions

New peptides identified from fermented milk were synthesized and analyzed in vitro and in a cellular model for their antioxidant properties. Four of these peptides, **N-15-M**, **E-11-F**, **Q-14-R** and **A-17-E**, were selected for their great protective effects against the action of oxidative stress induced by TbOOH both in the rescue of the viability and in the inhibition of ROS production. The selected bioactive peptides were further studied in order to better understand the mechanism of action of their antioxidant properties. The main conclusion, highlighted by the obtained results, was that the observed protective effects against oxidative stress of the antioxidant fermented milk-derived bioactive peptides were mostly due to the activation of Keap1/Nrf2 pathway.

## Figures and Tables

**Table 1:** Percentage of viability (MTT test) in Caco-2 cells in the presence of the isolated 5–30% and 30–50% acetonitrile (ACN) fractions. Means of at least three experiments (eight replicates for each experiment) were compared with the treated control. (* *p* < 0.05).

Fraction	Percentage of Cell Viability	
	None	TbOOH
Control	100	73.11 ± 8.22
5–30% ACN	103.59 ± 5.20	83.60 ± 2.06 *
30–50% ACN	108.67 ± 10.15	77.80 ± 3.27

**Table 2:** Sequences and characteristics of the synthesized peptides.

Native Protein	Peptide Sequence	Name	Number of Residues	Peptide Fragment	Monoisotopic MW (Da)	Presence on BIOPEP Database [36]
κ-casein	QYVLSRYPSYGIN	Q-13-N	13	50–62	1558.76	NO
	KYIPIQYVLS	K-10-S	10	45–54	1222.68	NO
	INNQFLPYPYYAKPA	I-15-A	15	72–86	1797.89	NO
	DKTEIPTINTIASGEPT	D-17-T	17	136–152	1785.88	YES (ID 8194—Kappacin; activity: Antibacterial)
	AVRSPAQILQWQ	A-12-Q	12	87–98	1395.75	NO
	VIESPPEINTVQ	V-12-Q	12	173–184	1324.67	YES (ID 8194—Kappacin; activity: Antibacterial)
	NTVPAKSCQAQPTTm *	N-15-M	15	102–116	1591.72	NO
β-casein	NVPGEIVESL	N-10-L	10	22–31	1055.53	YES (ID: 8173—peptide derived from bovine β-casein (1–28); activity: Immunomodulating)
	VYPFPGPIPN	V-10-N	10	74–83	1099.55	YES (ID: 7564 e 9240; ACE inhibitor; activity: ACE inhibitor)
	HKEMPFPKYPVEPFTESQ	H-18-Q	18	121–138	2190.03	NO
	SQSKVLPVPQKAVPYPQ	S-17-Q	17	181–197	1865.03	NO
	SWMHQPHQPLPPT	S-13-T	13	157–169	1554.72	NO
	VVPPFLQPE	V-9-E	9	98–106	1024.54	NO
	EDELQDKIHPF	E-11-F	11	57–67	1369.64	NO
	FPKYPVEPF	F-9-F	9	126–134	1122.56	NO
αS1-casein	APSFSDIPNPIGSENSE	A-17-E	17	191–207	1759.77	NO
	KHQGLPQEVLNENLL	K-15-L	15	22–36	1730.92	YES (ID: 8171—Isracidin-peptide derived from αS1-casein (1–23); activity: Immunomodulating)
	PFPEVFGKE	P-9-E	9	42–50	1048.51	NO
αS2-casein	QGPIVLNPWDQVKR	Q-14-R	14	116–129	1648.89	NO
	ALPQYLKTVYQHQK	A-14-K	14	190–203	1715.92	YES (ID: 8257, 8258, 8259; fragments of bovine αS2-casein; activity: Antibacterial)
	IQPKTKVIPYVRYL	I-14-L	14	209–222	1717.01	YES (ID: 8255; 8256; 8257; 8258; 8259; fragments of bovine αS2-casein; activity: Antibacterial)
	FLKKISQRYQKF	F-12-F	12	163–174	1584.90	YES (ID: Casocidin-I f(150–188); activity: antibacterial)
NaPi2B **	EKDDTGTPITKIELVPSH	E-18-H	18	36–53	1979.01	NO

*: peptide N-15-M was identified by Proteome Discoverer Software as NTVPAKSCQAQPTTm, with an oxidized Methionine at position 15, but it was synthesized with not oxidized Methionine; ** Sodium-dependent phosphate transport protein 2B.

**Table 3:** Evaluation of antioxidant properties of the identified peptides in vitro using 2,2′-azinobis(3-ethylbenzo-thiazoline 6-sulfonate) (ABTS) and 1,1-diphenyl-2-picrylhydrazyl (DPPH) scavenging tests and in Caco-2 cells pretreated with the 23 peptides. Oxidative stress was induced by 200 µM TbOOH.

Samples	In Vitro Antioxidant Tests		Percentage of Cell Viability	
	DPPH (%) Scavenging (a)	ABTS TEAC (b)	None (c)	TbOOH (d)
**Cnt**	n.d.	n.d.	100	73.11 ± 8.22
**Q-13 N**	24.44 ± 7.07	6.09 ± 1.08	104.10 ± 6.64	64.03 ± 10.15
**K-10-S**	19.09 ± 3.35	4.14 ± 0.82	97.27 ± 6.69	55.32 ± 10.01
**I-15-A**	19.40 ± 3.64	5.71 ± 1.09	104.37 ± 5.12	76.19 ± 13.27
**N-10-L**	13.19 ± 5.50	n. d.	119.52 ± 15.74	77.26 ± 12.44
**V-10-N**	12.24 ± 5.94	0.97 ± 0.48	110.89 ± 9.93	65.40 ± 17.60
**F-9-F**	12.69 ± 7.02	2.45 ± 0.54	109.47 ± 12.69	69.30 ± 12.78
**H-18-Q**	19.93 ± 5.13	1.30 ± 0.69	91.92 ± 8.99	52.61 ± 16.83
**S-13-T**	4.93 ± 3.45	2.96 ± 0.59	107.33 ± 12.39	71.60 ± 6.90
**S-17-Q**	3.74 ± 0.55	0.83 ± 0.36	101.47 ± 7.46	74.12 ± 11.76
**Q-14-R**	12.22 ± 4.72	1.28 ± 0.1	114.52 ± 15.55	81.47 ± 11.28
**A-17-E**	1.02 ± 0.15	0.64 ± 0.13	108.13 ± 13.19	78.33 ± 15.44
**D-17-T**	5.75 ± 0.38	0.84 ± 0.14	104.19 ± 5.62	73.36 ± 12.10
**E-18-H**	n. d.^a^	n. d.	103.64 ± 5.01	74.70 ± 10.52
**A-12-Q**	8.6± 5.98	1.39 ± 0.11	101.85 ± 4.69	78.89 ± 8.75
**V-12-Q**	n. d.	n. d.	112.70 ± 3.52	93.74 ± 10.68
**N-15-M**	16.93 ± 3.42	15.18 ± 0.04	107.99 ± 5.96	83.40 ± 5.51
**V-9-E**	n. d.	n. d.	106.59 ± 8.02	80.55 ± 6.62
**E-11-F**	n. d.	n. d.	111.51 ± 13.50	87.77 ± 4.50
**K-15-L**	4.14 ± 2.65	n. d.	104.70 ± 14.49	83.76 ± 6.30
**P-9-E**	n. d.	n. d.	100.97 ± 14.49	72.56 ± 3.68
**F-12-F**	15.29 ± 3.33	6.38 ± 0.29	97.73 ± 19.21	75.91 ± 4.80
**A-14-K**	2.16 ± 0.83	7.19 ± 0.23	84.39 ± 9.69	71.51 ± 5.22
**I-14-L**	22.9 ± 1.21	8.56 ± 0.43	92.98 ± 13.61	84.48 ± 8.64

^a^ n. d.: not detected with the used assay.

**Table 4:** Residues involved in the binding of Keap1 with Nrf2 or the analyzed peptides.

KEAP1	NRF2	A-17-E	Q-14-R	N-15-M
ARG 336		Glu 14		Thr 13
SER 363	Glu 82	Ser 3		
ARG 380	Glu 82		Val 5, Asn 7	Ala 5
ASN 382	Glu 82, Phe 83		Ile 4	Ala 5
ASN 387			Asn 7	
ARG 415	Glu 79, Thr 80	Ser 3	Gln 11	Asn 1
HIS 432			Asn 7	
GLY 433				Lys 6
ILE 435				Lys 6
SER 508	Glu 79		Gln 11	
GLY 509				Asn 1
TYR 525	Glu 79		Asp 10	
GLN 530	Glu 78		Arg 14	
TYR 572	Leu 76, Gly 81	Asn 9		Ala 10
HIS 575			Gly 2	
THR 576			Gln 1	Gln 11
SER 602	Thr 80			Thr 2

**Table 5:** Features of the studied peptides (bold) and their produced fragments, using RP-HPLC and MS analysis.

Peptide	Sequence	Retention Time (min)	Theoretical Mass	Measured MW	RP-HPLC Estimation AP (%)	RP-HPLC Estimation BL (%)
**N-15-M**	NTVPAKSCQAQPTTM	5.77	1575.73	1576.46	60.76 ± 8.83	0.13 ± 0.04
**V-13-M**	VPAKSCQAQPTTM	5.22	1360.63	1361.41	2.21 ± 0.53	n. d. ^a^
**A-11-M**	AKSCQAQPTTM	6.47	1164.51	1165.53	44.96 ± 9.09	0.16 ± 0.02
**S-9-M**	SCQAQPTTM	7.18	965.38	965.43	n. d.	0.12 ± 0.03
**E-11-F**	EDELQDKIHPF	7.63	1369.64	1370.56	88.28 ± 5.89	0.21 ± 0.01
**D-10-F**	DELQDKIHPF	6.75	1240.598	1241.42	4.17 ± 0.27	n. d.
**Q-14-R**	QGPIVLNPWDQVKR	8.73	1648.90	1649.60	87.59 ± 12.18	0.02 ± 0.01
**G-13-R**	GPIVLNPWDQVKR	8.29	1520.83	1521.76	3.19 ± 0.27	n. d.
**Q-13-K**	QGPIVLNPWDQVK	5.03	1492.79	1493.56	n. d.	0.07 ± 0.05
**I-11-R**	IVLNPWDQVKR	8.11	1366.76	1367.54	1.47 ± 0.19	n. d.
**Q-11-Q**	QGPIVLNPWDQ	7.19	1265.63	1266.94	n. d.	0.11 ± 0.01
**N-10-Q**	VLNPWDQVKR	7.28	1253.68	1254.60	7.44 ± 0.85	n. d.
**N-8-R**	NPWDQVKR	6.37	1041.52	1042.46	0.55 ± 0.15	n. d.
**A-17-E**	APSFSDIPNPIGSENSE	8.60	1759.80	1760.66	68.4 ± 4.6	0.05 ± 0.03
**A-16-S**	APSFSDIPNPIGSENS	6.73	1630.73	1630.45	3.22 ± 0.00	n. d.
**S-15-E**	SFSDIPNPIGSENSE	8.00	1591.69	1592.48	8.97 ± 0.53	0.30 ± 0.07
**F-14-E**	FSDIPNPIGSENSE	7.75	1504.65	1505.54	3.07 ± 0.60	n. d.

^a^ n. d.: not detected with RP-HPLC analysis.

## Supplementary Materials

The following are available online at https://www.mdpi.com/2076-3921/9/2/117/s1, Figure S1: Fraction 6 uptake from Caco-2 cell monolayer. Cells were incubated in the presence of fraction 6 (150 µg) and the analysis of Apical (AP) and basolateral (BL) compartments was performed at the indicated time. Figure S2: Alignments between the reference proteins and the peptides identified by Proteome Discoverer and Mascot search. Oxidized methionine residues are indicated in lowercase (m); #PSM values are indicated in brackets near to each peptide sequence. The alignments are reported separated for each reference protein. Table S1: Files from ORBITRAP analysis of fraction 6.
