# Supplementary material for: Identification of New Peptides from Fermented Milk Showing Antioxidant Properties: Mechanism of Action
Source: Antioxidants (Basel). 2020 Jan 29;9(2):117. doi: 10.3390/antiox9020117 (PMC7070694; doi:10.3390/antiox9020117)
Supplement: Supplementary file 1 [file antioxidants-09-00117-s001.pdf]

Supplementary Material

Identification of new peptides from fermented milk showing antioxidant properties: mechanism of action

Federica Tonolo[1a], Federico Fiorese [1a], Laura Moretto [1], Alessandra Folda [1], Valeria Scalcon [1], Alessandro Grinzato [1], Stefania Ferro [1], Giorgio Arrigoni [1], Alberto Bindoli [2], Emiliano Feller [3], Marco Bellamio [3], Oriano Marin[1*] and Maria Pia Rigobello [1*]

[1] Department of Biomedical Sciences, University of Padova, Padova, Italy.
[2] Institute of Neuroscience, CNR, Padova, Italy.
[3] Centrale del Latte di Vicenza S.p.A., Vicenza, Italy.
* Correspondence: mariapia.rigobello@unipd.it, oriano.marin@unipd.it;

Figure S1. Fraction 6 uptake from Caco-2 cell monolayer. Cells were incubated in the presence of fraction 6 (150 μg) and the RP-HPLC analysis of apical (AP) and basolateral (BL) compartments was performed at the indicated time (10′ and 120′, λ=220 nm).

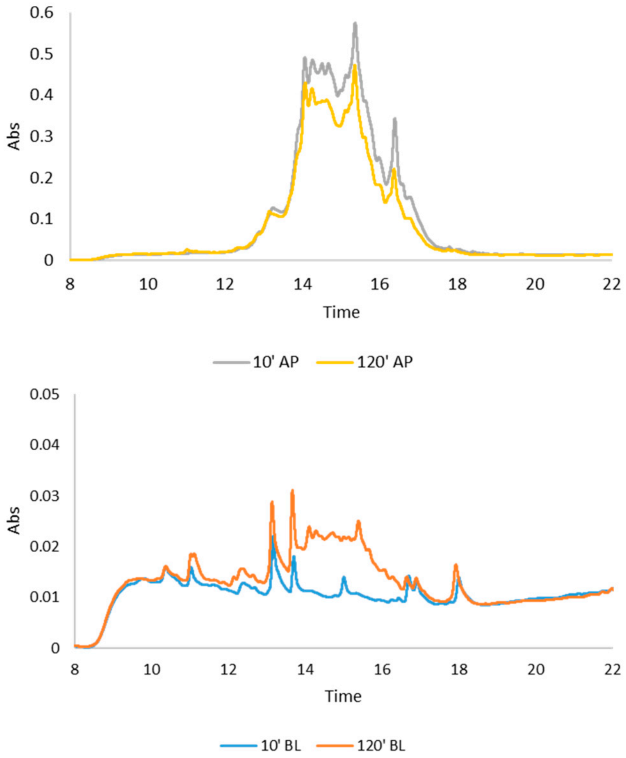

Figure S2. Alignments between the reference proteins and the peptides identified by Proteome Discoverer and Mascot search. Oxidized methionine residues are indicated in lowercase (m); #PSM values are indicated in brackets near to each peptide sequence. The alignments are reported separated for each reference protein.

>P02668 Kappa-casein

```
        10        20        30        40        50        60        70        80        90
MMKSFFLVVTILALTLPFLGAQEQNQEQPIRCEKDERFFSDKIAKYIPIQYVLSRYPSYGLNYYQQKPVALINNQFLPYPYYAKPAAVRSPAQILWQ
                                            KYIPIQYVLS(10)               NQFLPYPYYAKPA(22)
                                                 QYVLSRYPSYGIN(26)    INNQFLPYPYYAKPA(51)
                                            KYIPIQYVL (2)                 QFLPYPYYAKPA(19)
                                                     SRYPSYGLNYYQQKPV (2)
                                                   VLSRYPSYGLNYYQQKPV (2)
                                                      RYPSYGLNYYQQKPV (2)
                                                                     ALINNQFLPYPYYAKPA (2)
                                             YIPIQYVLS (1)
                                      FSDKIAKYIPI (1)
                                                    LSRYPSYGLNYYQQKPV (1)
                                                   VLSRYPSYGIN (1)
                                          IAKYIPIQYV (1)
                                          IAKYIPIQY (1)
                                                 QYVLSRYPSYGLNYYQQKPV (1)
                                          IAKYIPIQYVLS (1)
                                                                           FLPYPYYAKPA (1)
                                                                        NNQFLPYPYYAKPA (1)
                                                                           FLPYPYYAKPAAV (1)
                                                                          QFIPYPYYA (1)
                                                                         NQFLPYPYY (1)
                                                                          QFLPYPYYAKPAAVRSPA (1)
                                                                                   KPAAVRSPAQIL (1)
                                                                                 YAKPAAVRSPA(1)
                                                                                      AVRSPAQILWQ (1)

100       110       120       130       140       150       160       170       180       190
VLSNTVPAKSCQAQPTTMARHPHPHLSFMAIPPKKNQDKTEIPTINTIASGEPTSTPTTEAVESTVATLEDSPEVIESPPEINTVQVTSTAV
                   RHPHPHLSFM(12)                                         VIESPPEINTVQVTSTAV (1)
                TMARHPHPHLSFM(4)                                          VIESPPEINTVQ (1)

                                     DKTEIPTINTIASGEPT(7)
                TmARHPHPHLSFm (1)
                  ARHPHPHLSFMAIPPKKNQ (3)
                   RHPHPHLSFMAIPPKKNQ (3)
                   RHPHPHLSFm (3)    DKTEIPTINTIASGEPTSTPTTE (2)
```

DKTEIPTINTIASGEPTSTPTTEAVE (2)
QDKTEIPTINTIASGEPTSTPTTE (2)

HPHPHLSFm (2)
ARHPHPHLSFmAIPPKKNQ (2)
TMARHPHPHLSFm (2)
RHPHPHLSFmAIPPKKNQ (2)
LSFmAIPPKKNQ (1)

NTVPAKSCQAQPTTm (1)
SNTVPAKSCQAQPTTm (1)
ARHPHPHLSFmAIPPKKNQD (1)
DKTEIPTINTIASGEPTSTPT (1)
KTEIPTINTIASGEPT (1)
QDKTEIPTINTIASGEPT (1)
AIPPKKNQDKTEIPTINTIASGEPTSTPTTE (1)

P02666 Beta-casein

10 20 30 40 50 60 70 80 90 100 110

MKVLILACLVALALARELEELNVPGEIVESLSSSEESITRINKKIEKFQSEEQQQTEDELQDKIHPFAQTQSLVYPFPGPIPNSLPQNIPPLTQTPVVVPPFLQPEVMGV

NVPGEIVESL(8)
RELEELNVPGEIVE (1)
EQQQTEDELQDKIHPF (1)
QSEEQQQTEDELQDKIHPF (1)
EDELQDKIHPF (1)
SEEQQQTEDELQDKIHPF (1)
DELQDKIHPF (1)
ELQDKIHPFAQTQSL (1)
ELQDKIHPF (1)
VYPFPGPIPN(8)
NIPPLTQTPV (1)
VVPPFLQPE (2)

120 130 140 150 160 170 180 190 200 210 220

SKVKEAMAPKHKEMPFPKYPVEPFTESQSLTLTDVENLHLPLPLLQSWMHQPHQPLPPTVMFPPQSVLSLSQSKVLPVPQKAVPYPQRDMPIQAFLLYQEPVLGPVRGPF

HKEMPFPKYPVEPFTESQ(6)
HKEMPFPKYPVEPF (3)
KEMPFPKYPVEPF (2)
KEMPFPKYPVEPFTESQ (2)
EmPFPKYPVEPFTESQ (2)
PFPKYPVEPFTESQ (2)
KEmPFPKYPVEPF (2)
SWMHQPHQPLPPT(4)
SWmHQPHQPLPPT (1)
VmFPPQSVL(1)
SQSKVLPVPQKAVPYPQ(4)
SLSQSKVLPVPQKAVPYPQ (2)
SKVLPVPQKAVPYPQ (2)
LSQSKVLPVPQKAVPYPQ (2)
SLSQSKVLPVPQKAVPYPQR (2)

HKEmPFPKYPVEPFTESQ (2)
KEmPFPKYPVEPFTESQ (2)
HKEmPFPKYPVEPFTE (2)
EMPFPKYPVEPFTESQ (1)
EmPFPKYPVEPFTE (1)
FPKYPVEPFTESQ (1)
HKEmPFPKYPVEPFTES (1)
HKEmPFPKYPVEPF (1)
FPKYPVEPF (1)
HKEMPFPKYPVEPFTESQS (1)
HKEMPFPKYPVEPFTE (1)
HKEMPFPKYPVEPFTES (1)
EmPFPKYPVEPF (1)
EMPFPKYPVEPFTESQS (1)
PFPKYPVEPFTE (1)
EmPFPKYPVEPFTES (1)
PFPKYPVEPFTESQS (1)
MAPKHKEMPFPKYPVEPF (1)
MAPKHKEmPFPKYPVEPF (1)

KVLPVPQKAVPYPQR (2)
SQSKVLPVPQKAVPYPQR (2)
KVLPVPQKAVPYPQ (2)
VLSLSQSKVLPVPQ (1)
SLSQSKVLPVPQ (1)
KVLPVPQKAVPYPQRD (1)

PIIV

>P02662 Alpha-S1-casein

10 20 30 40 50 60 70 80 90 100 110

MKLLILTCLVAVALARPKHPIKHQGLPQEVLNENLLRFFVAPFPEVFGKEKVNELSKDIGSESTEDQAMEDIKQMEAESISSSEEIVPNSVEQKHIQKEDVPSERYLGYL

KHQGLPQEVLNENLL(4)
HQGLPQEVLNENLL (1)
VAPFPEVFGKE (1)
APFPEVFGKE (1)
VAPFPEVFGKEK (1)
PFPEVFGKE (1)
VAPFPEVF (1)
VAPFPEVFGK (1)
FFVAPFPE (1)
DVPSERYLGYL(1)

120 130 140 150 160 170 180 190 200 210

EQLLRLKKYKVPQLEIVPNSAEERLHSMKEGIHAQQKEPMIGVNQELAYFYPELFRQFYQLDAYPSGAAWYYVPLGTQYTDAPSFSDIPNPIGSENSEKTTMPLW

KYKVPQLEIVPN (1)
KVPQLEIVPNSAEERLH (1)
QKEPMIGVNQELAY (1)
QKEPmIGVNQELAY (1)
APSFSDIPNPIGSENSE(48)
APSFSDIPNPIGSE(5)
GTQYTDAPSFSDIPNPIGSENSE(4)
TDAPSFSDIPNPIGSENSE (2)
GTQYTDAPSFSDIPNPIGSENSEK (2)
APSFSDIPNPIGSEN (2)

```
                                                                                          APSFSDIPNPIGSENSEKT (2)
                                                                                       YTDAPSFSDIPNPIGSENSE (1)
                                                                                    GTQYTDAPSFSDIPNPIGSE (1)
                                                                                        TDAPSFSDIPNPIGSENSEK (1)
                                                                                      QYTDAPSFSDIPNPIGSENSEK (1)
                                                                                         DAPSFSDIPNPIGSENSE (1)
                                                                                          APSFSDIPNPIGSENSEK (1)
                                                                                         DAPSFSDIPNPIGSENSEK (1)
                                                                                      QYTDAPSFSDIPNPIGSE (1)
                                                                                      QYTDAPSFSDIPNPIGSENSE (1)
                                                                                     TQYTDAPSFSDIPNPIGSENSE (1)
                                                                                                IPNPIGSENSEKTTMP(1)
                                                                                              SDIPNPIGSENSEKTTMPLW(1)
                                                                                             FSDIPNPIGSENSEKTTmP (1)
                                                                                                IPNPIGSENSEKTTmPLW(1)
                                                                                                IPNPIGSENSEKTTMPLW(1)
                                                                                             FSDIPNPIGSENSEKTTMP (1)
                                                                                         DAPSFSDIPNPIGSE (1)
```

>P02663 Alpha-S2-casein

```
         10        20        30        40        50        60        70        80        90        100       110
MKFFIFTCLLAVALAKNTMEHVSSSEESIISQETYKQEKNMAINPSKENLCSTFCKEVVRNANEEEYSIGSSSEESAEVATEEVKITVDDKHYQKALNEINQFYQKFPQY

         120       130       140       150       160       170       180       190       200       210       220
LQYLYQGPIVLNPWDQVKRNAVPITPTLNREQLSTSEENSKKTVDMESTEVFTKKTKLTEEEKNRLNFLKKISQRYQKFALPQYLKTVYQHQKAMKPWIQPKTKVIPYVRYL
     QGPIVLNPWDQVKR(3)                                  TKKTKLTEEEKNRLNFLK (1)   ALPQYLKTVYQHQK (2)
     QGPIVLNPWDQVKRN(2)                                                FLKKISQRYQKF (1)
     QGPIVLNPWDQVK (1)                                                             PQYLKTVYQHQK (1)
    YQGPIVLNPWDQVKRN (1)                                                         ALPQYLKTVYQHQKA (1)
                    AVPITPTLNREQL(1)                                                           AMKPWIQPK (1)
                  RNAVPITPTLNREQL (1)                                                               IQPKTKVIPYVRYL(1)
```

Other identified peptides

>Q27960 Sodium-dependent phosphate transport protein 2B

10 20 30 40 50 60 70 80 90 100 110
MAPWPELENSQPTSEKYTVKADGEQSAKPEKAKETEKDDTGTPITKIELVPSHSTATLIEEPTEVEDPWDLPELKDTGLKWSERDTKGKILCVFQGIGKFILLLVFLYFF
EKDDTGTPITKIELVPSH (3)
DTGTPITKIELVPSH (2)
KDDTGTPITKIELVPSH (1)
DDTGTPITKIELVPSH (1)

120 130 140 150 160 170 180 190 200 210 220
VCSLDVLSSAFQLVGGKVAGKFFNNNSIMSNPLAGMVIGVLVTVLVQSSSTSTSIVVSMVASSLLPVHAAIPIIMGANIGTSITNTIVALMQAGDRKEFRRAFAGATVHD

230 240 250 260 270 280 290 300 310 320 330
FFNWLSVLVLLPLEAATGYLERLTNLVVESFHFKNGEEAPELLKVITDPFTKLIIQLDKSILNQIAMNDESVQNKSMIKIWCKTFTNVTERNVTVPSPENCTSPSLCWTD

340 350 360 370 380 390 400 410 420 430 440
GLYTWTIKNVTYKENIAKCQHIFVNFNLSDAIVGTILLITSLLILCTCLILIVKLLGSVLRGQVAAVIKKTINTDFPYPFSWVTGYLAILVGAGMTFIVQSSSVFTSAMT

450 460 470 480 490 500 510 520 530 540 550
PLIGIGVISIQRAYPLTLGANIGTTTTAILAALASPGSTLKSSLQIALCHFFFNISGIILWYPIPFTRLPIRLAKGLGNISSKYRWFAIVYLIVFFLLIPLAVFGLSLIG

560 570 580 590 600 610 620 630 640 650 660
WPVLVGVASPIVLVILLVVVLKILQSFCPGSLPQKLRSWDFLPFWMRSLEPWDKLITSLTSCFQMRCCCCCRVCCRLCCGLCGCSKCCRCTKCSEDLEEGKDEPVKSPEA

670 680 690
FNNLAMDKEAQDGVTKSEVDASGTKIVSSVTAL

>P81265-2 Isoform Short of Polymeric immunoglobulin receptor

10 20 30 40 50 60 70 80 90 100 110
MSRLFLACLLAIFPVVSMKSPIFGPEEVTSVEGRSVSIKCYYPPTSVNRHTRKYWCRQGAQGRCTTLISSEGYVSDDYVGRANLTNFPESGTFVVDISHLTHKDSGRYKC

120 130 140 150 160 170 180 190 200 210 220
GLGISSRGLNFDVSLEVSEETAIPASPSVVKGVRGGSVTVSCPYNPKDANSAKYWCHWEEAQNGRCPRLVESRGLIKEQYEGRLALLTEPGNGTYTVILNQLTDQDTGFY

230 240 250 260 270 280 290 300 310 320 330
WCVTDGDTRWISTVELKVVQGEPSLKVPKNVTAWLGEPLKLSCHFPCKFYSFEKYWCKWSNRGCSALPTQNDGPSQAFVSCDQNSQVVSLNLDTVTKEDEGWYWCGVKEG

340 350 360 370 380 390 400 410 420 430 440
PRYGETAAVYVAVESRVKGSQGAKQVKAAPAGAAIQSRAGEIQNKALLDPSFFAKESVKDAAGGPGAPADPGRPTGYSGSSKALVSTLVPLALVLVAGVVAIGVVRARHR
FFAKESVKDAAGGPGAPADPGRPT (2)
DAAGGPGAPADPGRPT (1)
SVKDAAGGPGAPADPGRPT (1)

AKESVKDAAGGPGAPADPGRPT (1)

450 460 470 480 490 500 510 520 530

KNVDRISIRSYRTDISMSDFENSRDFEGRDNMGASPEAQETSLGGKDEFATTTEDTVESKEPKKAKRSSKEEADEAFTTFLLQAKNLASAATQNGPTEA

>P80195 Glycosylation-dependent cell adhesion molecule

10 20 30 40 50 60 70 80 90 100 110

MKFLCVLLLASLAATSLAILNKPEDETHLEAQPTDASAQFIRNLQISNEDLSKEPSISREDLISKEQIVIRSSRQPQSQNPKLPLSILKEKHLRNATLGSEETTEHTPSD

DASAQFIRNL (3) DLISKEQIVIR (1) SQNPKLPLSILK (1)

DASAQFIRNLQISNE (1)

120 130 140 150

ASTTEGKLMELGHKIMRNLENTVKETIKYLKSLFSHAFEVVKT

>C6KGD8 Histatherin

10 20 30 40 50

MKIFIFIFIMALILAMIRADSSEEKRHRKRKKHHRGYFQQYQPYQRYPLNYPPAYPFP

NYPPAYPFP (3)

>A5GT44 3-phosphoshikimate 1-carboxyvinyltransferase (Synechococcus sp (strain RCC307)

10 20 30 40 50 60 70 80 90 100 110

MGSVCLSGGGSLHGSVRVPGDKSISHRALLFGAIATGTTTIEGLLPAEDPLSTAACLRAMGVTVSAIDSSGVVRVEGVGLDGLQEPAQVLDCGNSGTTMRLMLGLLAGRH

120 130 140 150 160 170 180 190 200 210 220

RHGRHFVLDGDGSLRRRPMARVAQPLAQMGAEIGGREGGNKAPLAIAGQTLSGGTIRTPVASAQVKSALLLAGLTAKGSTTVIEPALSRDHSERMLRAFGAELISEPHAA

230 240 250 260 270 280 290 300 310 320 330

EGPTAIVRPGAELHGQHVVVPGDISSAAFWLIAALVVPGTELTIENVGINPTRTGILDVLEQMGAPLEVLNQRDVAGEPVADLRVRYSPLKAFEIGGELIPRLVDEIPIL

mGAPLEVLNQRDV (1)

340 350 360 370 380 390 400 410 420 430

SVAALCAEGTSVMRDAAELRVKETDRLAVMARQLRAMGAELEETTDGMVIPGGQRLTGAQVDSETDHRVAMSLAVAALIASGDTSIDQSEAAAVSYPSFWDELARLQRS

Table S1: Files from ORBITRAP analysis of fraction 6

A2	Sequence	# PSMs	# Proteins	# Protein Groups	Protein Group Accessions	Modifications	ΔCn	q-Value	PEP	IonScore	Exp Value	Charge	MH+ [Da]	ΔM [ppm]	RT [min]
High	VAPFPEVF	1	1	1	P02662		0.0000	0.007	0.04344	33	9.681784003	2	905.47649	-0.32	24.78
High	FFVAPFPE	1	1	1	P02662		0.0000	0.007	0.09821	27	28.06770869	2	953.47643	-0.37	24.47
High	VMFPPQSVL	1	1	1	P02666		0.0000	0.007	0.04964	45	0.494577975	2	1017.54424	0.41	24.07
High	VVPPFLQPE	2	1	1	P02666		0.0000	0	0.000363	57	0.018245656	2	1025.56719	0.51	22.50
High	VmFPPQSVL	1	1	1	P02666	M2(Oxidation)	0.0000	0.007	0.04304	39	2.451581831	2	1033.53874	0.01	23.38
High	PFPEVFGKE	1	1	1	P02662		0.0000	0	0.008823	35	5.636172121	2	1049.53154	1.20	21.75
High	NVPGEIVESL	8	1	1	P02666		0.0000	0	0.0004486	57	0.021863805	2	1056.55779	0.54	23.16
High	NYPPAYPFP	3	1	1	C6KGD8		0.0000	0.002	0.01853	48	0.249956019	2	1065.50432	0.27	23.22
High	NIPPLTQTPV	1	1	1	P02666		0.0000	0.002	0.01827	40	0.696529906	2	1079.60979	0.19	20.71
High	VAPFPEVFGK	1	1	1	P02662		0.0000	0.006	0.03205	29	20.21645871	2	1090.59331	0.08	21.87
High	YIPIQYVLS	1	2	1	P02668		0.0000	0	0.004535	40	0.964035887	2	1095.60918	0.63	24.39
High	AMKPWIQPK	1	1	1	P02663		0.0000	0.002	0.01865	28	14.82810541	3	1098.61274	-0.16	17.49
High	VYPFPGPIPN	8	1	1	P02666		0.0000	0	0.0001534	71	0.001171555	2	1100.57817	0.56	22.53
High	IAKYIPIQY	1	1	1	P02668		0.0000	0.002	0.01052	46	0.162868872	2	1108.64043	0.27	20.24
High	HPHPHLSFm	2	1	1	P02668	M9(Oxidation)	0.0000	0	0.005337	36	2.599880153	2	1118.52031	0.24	16.46
High	APFPEVFGKE	1	1	1	P02662		0.0000	0	0.003675	51	0.152771097	2	1120.56755	0.13	21.04
High	FPKYPVEPF	1	1	1	P02666		0.0000	0	0.002009	40	1.516676114	2	1123.58293	0.56	21.70
High	ELQDKIHPF	1	1	1	P02666		0.0000	0.007	0.07196	27	30.68032997	2	1126.58940	0.20	18.50
High	YAKPAAVRSPA	1	1	1	P02668		0.0000	0.002	0.01662	26	45.99496154	3	1130.63147	-0.22	14.83
High	DASAQFIRNL	3	1	1	P80195		0.0000	0	0.0003083	59	0.024519501	2	1134.58940	-0.75	21.96
High	KYIPIQYVL	2	2	1	P02668		0.0000	0.007	0.0573	28	7.168561998	2	1136.67070	-0.64	28.46
High	NVPGEIVESLS	1	1	1	P02666		0.0000	0	0.0001116	47	0.376050697	2	1143.58940	0.13	21.94
High	QFIPYPYYA	1	1	1	P02668		0.0000	0.002	0.02127	53	0.067122276	2	1161.56291	1.19	23.68
High	VPPFLQPEVm	1	1	1	P02666	M10(Oxidation)	0.0000	0	0.001174	49	0.230114459	2	1172.60210	0.02	22.08
High	NQFLPYPYY	1	1	1	P02668		0.0000	0	0.002655	41	0.95995991	2	1204.56877	1.19	23.69

High	IAKYIPIQYV	1	1	1	P02668		0.0000	0.006	0.02882	47	0.095405094	2	1207.70952	0.80	21.51
High	VAPFPEVFGKE	1	1	1	P02662		0.0000	0	0.000009952	55	0.061501386	2	1219.63640	0.47	21.67
High	KYIPIQYVLS	10	2	1	P02668		0.0000	0.002	0.01459	51	0.050206481	2	1223.70464	0.96	23.25
High	DELQDKIHPF	1	1	1	P02666		0.0000	0.006	0.02988	32	9.675319894	2	1241.61650	0.30	19.90
High	KPAAVRSPAQIL	1	1	1	P02668		0.0000	0	0.002528	45	0.109924736	2	1250.75871	0.57	18.18
High	RHPHPHLSFM	12	1	1	P02668		0.0000	0	0.006897	22	98.70954218	3	1258.62641	0.12	27.62
High	VLSRYPSYGIN	1	1	1	P02668		0.0000	0	0.00002328	53	0.080212077	2	1268.66460	0.95	18.69
High	RHPHPHLSFm	3	1	1	P02668	M10(Oxidation)	0.0000	0.002	0.01456	26	40.32938131	2	1274.62212	0.75	15.59
High	SLSQSKVLPVPQ	1	1	1	P02666		0.0000	0.007	0.04149	40	0.846810255	2	1282.73735	0.60	18.78
High	FSDKIAKYIPI	1	1	1	P02668		0.0000	0	0.0001275	61	0.005241955	2	1294.74126	0.51	21.82
High	DVPSERYLGYL	1	1	1	P02662		0.0000	0	0.000002605	65	0.004774921	2	1311.65874	0.58	22.34
High	DLISKEQIVIR	1	1	1	P80195		0.0000	0.002	0.01731	53	0.038589215	2	1313.78044	1.26	19.42
High	VIESPPEINTVQ	1	1	1	P02668		0.0000	0	0.001445	48	0.242440168	2	1325.69487	0.07	19.35
High	FLPYPYYAKPA	1	1	1	P02668		0.0000	0	0.000001573	69	0.002786347	2	1329.68840	0.44	20.61
High	SQNPKLPLSILK	1	1	1	P80195		0.0000	0	0.001103	44	0.123173071	3	1337.81495	-0.17	20.16
High	VAPFPEVFGKEK	1	1	1	P02662		0.0000	0	0.0002119	46	0.427583453	2	1347.73149	0.52	19.99
High	EDELQDKIHPF	1	1	1	P02666		0.0000	0.002	0.02433	36	4.133464822	2	1370.66008	0.99	19.46
High	LSFmAIPPKKNQ	1	2	1	P02668	M4(Oxidation)	0.0000	0.006	0.0325	39	1.942695636	3	1389.75721	0.90	17.64
High	LSLSQSKVLPVPQ	1	1	1	P02666		0.0000	0	0.0009985	52	0.03995823	2	1395.82207	1.02	20.31
High	AVRSPAQILQWQ	1	2	1	P02668		0.0000	0.007	0.09472	35	3.725904626	2	1396.77117	1.11	21.62
High	DAAGGPGAPADPGRPT	1	2	1	P81265-2		0.0000	0	0.0002422	50	0.141333489	2	1406.66667	0.49	15.07
High	IAKYIPIQYVLS	1	1	1	P02668		0.0000	0	0.002541	40	0.498879976	2	1407.82598	0.94	23.33
High	KYKVPQLEIVPN	1	1	1	P02662		0.0000	0.002	0.02045	32	5.407702321	2	1427.82707	0.94	19.75
High	APSFSDIPNPIGSE	5	1	1	P02662		0.0000	0	0.00003667	65	0.004862255	2	1430.68157	1.18	22.56
High	VLPVPQKAVPYPQ	1	1	1	P02666		0.0000	0.002	0.01873	44	0.241754283	2	1435.83379	2.07	19.40
High	PFPKYPVEPFTE	1	1	1	P02666		0.0000	0.006	0.03233	32	11.49529799	2	1450.72673	0.95	21.57
High	AVPITPTLNREQL	1	1	1	P02663		0.0000	0	0.001817	37	1.743858157	2	1451.82329	1.08	20.03
High	mGAPLEVLNQRDV	1	1	1	A5GT44	M1(Oxidation)	0.0000	0.007	0.06658	48	0.291337664	2	1457.74639	3.16	29.00
High	QFLPYPYYAKPA	19	1	1	P02668		0.0000	0	0.008801	37	3.858805578	2	1457.74797	1.08	27.33

High	KmEGSPYLFLTR	2	0	0		M2(Oxidation)	0.0000	0	0.008965	41	1.601226974	2	1457.74797	1.51	27.88
High	QGPIVLNPWDQVK	1	1	1	P02663		0.0000	0	0.00000351	52	0.089759934	2	1493.81206	0.60	21.95
High	VLSLSQSKVLPVPQ	1	1	1	P02666		0.0000	0	1.385E-07	96	1.10065E-06	2	1494.89092	1.23	20.92
High	EmPFPKYPVEPF	1	1	1	P02666	M2(Oxidation)	0.0000	0.002	0.01936	34	6.670055297	2	1496.71453	0.96	22.19
High	FLPYPYYAKPAAV	1	1	1	P02668		0.0000	0	0.000002163	61	0.013279052	2	1499.79460	0.83	21.56
High	PQYLKTVYQHQK	1	1	1	P02663		0.0000	0	0.001189	38	3.403534579	3	1532.82218	0.11	18.60
High	APSFSDIPNPIGSEN	2	1	1	P02662		0.0000	0	0.0002071	58	0.020134245	2	1544.72429	0.96	21.99
High	DAPSFSDIPNPIGSE	1	1	1	P02662		0.0000	0.002	0.01822	29	13.58679761	2	1545.70806	0.80	22.95
High	SWMHQPHQPLPPT	4	1	1	P02666		0.0000	0	0.006232	28	23.49431909	2	1555.74907	1.01	19.13
High	QYVLSRYPSYGIN	26	1	1	P02668		0.0000	0	0.000000134	76	0.000465059	2	1559.78752	1.43	20.10
High	TMARHPHPHLSFM	4	1	1	P02668		0.0000	0	0.0004658	41	1.485955088	2	1561.75286	0.83	17.27
High	KVLPVPQKAVPYPQ	2	1	1	P02666		0.0000	0	0.001458	39	0.488186096	2	1563.92729	0.96	18.53
High	FPKYPVEPFTESQ	1	1	1	P02666		0.0000	0	0.0006286	43	0.813015293	2	1568.76506	1.19	20.96
High	SWmHQPHQPLPPT	1	1	1	P02666	M3(Oxidation)	0.0000	0.007	0.06195	29	16.991181	2	1571.74602	2.29	17.05
High	NQFLPYPYYAKPA	22	1	1	P02668		0.0000	0	0.001325	62	0.012624502	2	1571.79167	1.50	21.18
High	TMARHPHPHLSFm	2	1	1	P02668	M13(Oxidation)	0.0000	0	0.00578	28	25.024367	3	1577.74692	0.29	16.01
High	FLKKISQRYQKF	1	1	1	P02663		0.0000	0	0.00375	17	164.235699	3	1585.92258	0.77	16.80
High	NTVPAKSCQAQPTTm	1	2	1	P02668	M15(Oxidation)	0.0000	0.007	0.0854	29	16.06211266	2	1592.74211	0.84	14.43
High	TmARHPHPHLSFm	1	1	1	P02668	M2(Oxidation); M13(Oxidation)	0.0000	0	0.0009288	24	52.40060137	2	1593.74309	1.07	15.63
High	QTEDELQDKIHPF	1	1	1	P02666		0.0000	0.002	0.01341	44	0.690315244	2	1599.76799	1.88	19.42
High	HQGLPQEVLNENLL	1	1	1	P02662		0.0000	0	1.367E-07	56	0.052739312	2	1603.84538	0.92	22.46
High	DTGTPITKIELVPSH	2	1	1	Q27960		0.0000	0	0.0002667	62	0.010977675	2	1607.86565	1.04	20.23
High	KEMPFPKYPVEPF	2	1	1	P02666		0.0000	0	0.000001768	65	0.007184984	2	1608.81560	1.53	21.65
High	QKEPMIGVNQELAY	1	1	1	P02662		0.0000	0.002	0.01408	34	7.856044125	2	1619.81120	0.84	20.20
High	KEmPFPKYPVEPF	2	1	1	P02666	M3(Oxidation)	0.0000	0	0.0005162	36	4.725748237	2	1624.80925	0.74	20.63
High	RELEELNVPGEIVE	1	1	1	P02666		0.0000	0	0.0006298	33	9.342385974	2	1625.83757	-0.37	21.05
High	QKEPmIGVNQELAY	1	1	1	P02662	M5(Oxidation)	0.0000	0	0.005551	31	16.84003579	2	1635.80693	1.33	19.44
High	QGPIVLNPWDQVKR	3	1	1	P02663		0.0000	0	0.006859	39	1.678679751	3	1649.91245	0.10	20.85

High	SKVLPVPQKAVPYPQ	2	1	1	P02666		0.0000	0	0.0001268	60	0.00532847	2	1650.95964	1.10	18.45
High	PFPKYPVEPFTESQ	2	1	1	P02666		0.0000	0	0.00003189	46	0.507442495	2	1665.81804	1.25	21.44
High	KTEIPTINTIASGEPT	1	1	1	P02668		0.0000	0	0.00001839	68	0.002982272	2	1671.88176	1.03	20.23
High	SNTVPAKSCQAQPTTm	1	1	1	P02668	M16(Oxidation)	0.0000	0	0.001589	38	1.871575095	2	1679.77385	0.62	14.55
High	NNQFLPYPYYAKPA	1	1	1	P02668		0.0000	0	0.0003465	66	0.004760289	2	1685.83440	1.27	21.14
High	DASAQFIRNLQISNE	1	1	1	P80195		0.0000	0	0.009294	33	10.4489446	2	1705.85320	1.60	21.75
High	EMPFPKYPVEPFTE	1	1	1	P02666		0.0000	0	0.00007683	52	0.099176549	2	1710.81035	1.11	23.20
High	IPNPIGSENSEKTTMP	1	1	1	P02662		0.0000	0	0.001597	55	0.05505082	2	1714.83476	1.78	19.20
High	ALPQYLKTVYQHQK	2	1	1	P02663		0.0000	0	0.00004255	35	5.973183075	2	1716.94475	0.90	18.61
High	IQPKTKVIPYVRYL	1	1	1	P02663		0.0000	0	0.003324	23	14.84178408	3	1718.03812	1.01	19.72
High	KVLPVPQKAVPYPQR	2	1	1	P02666		0.0000	0	0.000000198	51	0.019888432	3	1720.02903	1.23	17.37
High	SVKDAAGGPGAPADPGRPT	1	2	1	P81265-2		0.0000	0	0.0007104	40	2.378838569	3	1720.86087	-0.31	14.96
High	RNAVPITPTLNREQL	1	1	1	P02663		0.0000	0.002	0.02143	26	25.64301531	3	1721.96628	0.30	19.14
High	DDTGTPITKIELVPSH	1	1	1	Q27960		0.0000	0	0.005703	36	6.034282069	2	1722.89385	1.69	20.44
High	EmPFPKYPVEPFTE	1	1	1	P02666	M2(Oxidation)	0.0000	0	0.00001866	59	0.016794492	2	1726.80693	2.07	21.76
High	KHQGLPQEVLNENLL	4	1	1	P02662		0.0000	0	0.0009916	54	0.080386833	2	1731.94036	0.85	21.38
High	HKEMPFPKYPVEPF	3	1	1	P02666		0.0000	0	0.00002948	53	0.112941863	2	1745.87505	1.72	21.41
High	PFPKYPVEPFTESQS	1	1	1	P02666		0.0000	0.007	0.03796	27	40.26355345	2	1752.85027	1.30	21.09
High	ELQDKIHPFAQTQSL	1	1	1	P02666		0.0000	0.006	0.03216	31	15.7372164	2	1754.90972	1.41	19.59
High	APSFSDIPNPIGSENSE	48	1	1	P02662		0.0000	0	2.29E-09	122	6.40415E-09	2	1760.80120	2.14	22.18
High	KAVPYPQRDMPIQAF	1	1	1	P02666		0.0000	0	0.0003921	44	0.91740535	2	1760.91850	1.81	20.10
High	HKEmPFPKYPVEPF	1	1	1	P02666	M4(Oxidation)	0.0000	0	0.002265	42	1.34063127	2	1761.87029	1.88	19.63
High	QGPIVLNPWDQVKRN	2	1	1	P02663		0.0000	0	0.0002236	50	0.154496093	2	1763.95818	1.68	20.56
High	QSKVLPVPQKAVPYPQ	1	1	1	P02666		0.0000	0	0.00000367	49	0.086859637	2	1779.01933	1.65	18.45
High	DKTEIPTINTIASGEPT	7	1	1	P02668		0.0000	0	4.218E-07	88	3.68557E-05	2	1786.90837	0.78	20.61
High	ALPQYLKTVYQHQKA	1	1	1	P02663		0.0000	0	0.00006067	20	161.7685963	3	1787.97928	-0.58	18.80
High	INNQFLPYPYYAKPA	51	1	1	P02668		0.0000	0	1.554E-07	64	0.008685841	2	1798.91863	1.28	22.83
High	EmPFPKYPVEPFTES	1	1	1	P02666	M2(Oxidation)	0.0000	0.002	0.01649	30	11.64320474	2	1813.83769	1.27	21.56
High	KVLPVPQKAVPYPQRD	1	1	1	P02666		0.0000	0	0.009905	28	11.44732592	3	1835.05661	1.50	17.50

High	KDDTGTPITKIELVPSH	1	1	1	Q27960		0.0000	0	0.00006694	43	1.016800172	3	1850.98697	0.58	19.25
High	SQSKVLPVPQKAVPYPQ	4	1	1	P02666		0.0000	0	0.001452	64	0.003795117	2	1866.05193	1.87	18.44
High	DELQDKIHPFAQTQSL	1	1	1	P02666		0.0000	0	0.00001365	51	0.166747456	2	1869.93596	0.95	20.45
High	DAPSFSDIPNPIGSENSE	1	1	1	P02662		0.0000	0	2.001E-11	95	2.26752E-06	2	1875.82671	1.24	22.47
High	RYPSYGLNYYQQKPV	2	1	1	P02668		0.0000	0	0.0000329	68	0.003589147	2	1875.94060	0.94	18.91
High	VIESPPEINTVQVTSTAV	1	1	1	P02668		0.0000	0	3.783E-07	76	0.000461441	2	1883.99956	1.82	21.50
High	APSFSDIPNPIGSENSEK	1	1	1	P02662		0.0000	0	6.262E-11	84	7.0825E-05	2	1888.89556	1.67	20.77
High	QDKTEIPTINTIASGEPT	1	1	1	P02668		0.0000	0	0.000003218	57	0.048112271	2	1914.96880	1.69	20.38
High	EMPFPKYPVEPFTESQ	1	1	1	P02666		0.0000	0	2.122E-08	71	0.001329051	2	1925.90178	1.41	22.68
High	YQGPIVLNPWDQVKRN	1	1	1	P02663		0.0000	0.002	0.02465	40	2.378510439	3	1927.01841	-0.07	21.32
High	QYTDAPSFSDIPNPIGSE	1	1	1	P02662		0.0000	0	3.559E-10	76	0.000260406	2	1937.87822	0.94	23.16
High	EmPFPKYPVEPFTESQ	2	1	1	P02666	M2(Oxidation)	0.0000	0	0.0009749	47	0.260367133	2	1941.89678	1.44	21.43
High	KVPQLEIVPNSAEERLH	1	1	1	P02662		0.0000	0.007	0.0794	31	15.45876938	4	1959.06630	0.21	19.19
High	SRYPSYGLNYYQQKPV	2	1	1	P02668		0.0000	0	1.265E-07	77	0.000430803	2	1962.97368	1.44	19.03
High	HKEMPFPKYPVEPFTE	1	1	1	P02666		0.0000	0	0.008044	37	4.306350315	3	1975.96341	0.55	20.32
High	TDAPSFSDIPNPIGSENSE	2	1	1	P02662		0.0000	0	1.036E-11	95	2.14939E-06	2	1976.87468	1.33	22.32
High	LSQSKVLPVPQKAVPYPQ	2	1	1	P02666		0.0000	0	2.878E-08	54	0.030617674	2	1979.13432	0.92	19.01
High	EKDDTGTPITKIELVPSH	3	1	1	Q27960		0.0000	0	0.000007084	45	0.647015651	2	1980.03069	1.10	19.32
High	ALINNQFLPYPYYAKPA	2	1	1	P02668		0.0000	0	0.0007376	40	2.354510149	2	1983.04057	1.54	23.36
High	EQQQTEDELQDKIHPF	2	1	1	P02666		0.0000	0	0.000458	50	0.144092199	2	1984.92864	1.96	19.61
High	APSFSDIPNPIGSENSEKT	2	1	1	P02662		0.0000	0	0.0003209	43	0.879975984	2	1989.94304	1.49	20.90
High	HKEmPFPKYPVEPFTE	2	1	1	P02666	M4(Oxidation)	0.0000	0.006	0.03093	20	207.1052727	3	1991.95969	1.23	19.47
High	DAPSFSDIPNPIGSENSEK	1	1	1	P02662		0.0000	0	1.327E-10	83	6.81867E-05	2	2003.92266	1.65	21.31
High	EMPFPKYPVEPFTESQS	1	1	1	P02666		0.0000	0	0.0002746	32	7.867598157	2	2012.93266	0.78	22.66
High	IPNPIGSENSEKTTMPLW	1	1	1	P02662		0.0000	0	0.00001406	41	1.857835877	2	2013.99834	1.61	23.21
High	SQSKVLPVPQKAVPYPQR	2	1	1	P02666		0.0000	0	0.00004905	48	0.154356706	3	2022.15232	1.37	17.63
High	IPNPIGSENSEKTTmPLW	1	1	1	P02662	M15(Oxidation)	0.0000	0	0.00006641	47	0.403536513	2	2029.99321	1.57	21.64
High	QFLPYPYYAKPAAVRSPA	1	1	1	P02668		0.0000	0.007	0.06303	26	53.92282528	3	2039.07676	0.87	20.48
High	AKESVKDAAGGPGAPADPGRPT	1	2	1	P81265-2		0.0000	0.006	0.02788	29	33.47695459	3	2049.03769	0.78	14.82

High	KEMPFPKYPVEPFTESQ	2	1	1	P02666		0.0000	0	0.000001029	50	0.216684019	2	2053.99639	1.15	20.98
High	HKEMPFPKYPVEPFTES	1	1	1	P02666		0.0000	0	0.00002138	36	5.515215351	2	2062.99614	0.86	20.26
High	FSDIPNPIGSENSEKTTMP	1	1	1	P02662		0.0000	0	0.000000506	40	1.582327122	2	2063.96221	1.50	20.79
High	SLSQSKVLPVPQKAVPYPQ	2	1	1	P02666		0.0000	0	0.00003065	65	0.003231622	2	2066.16826	1.80	19.23
High	KEmPFPKYPVEPFTESQ	2	1	1	P02666	M3(Oxidation)	0.0000	0.002	0.02514	32	12.28548485	3	2069.99118	1.08	20.12
High	LSRYPSYGLNYYQQKPV	1	1	1	P02668		0.0000	0	6.538E-07	53	0.122597963	3	2076.05509	0.08	19.30
High	HKEmPFPKYPVEPFTES	1	1	1	P02666	M4(Oxidation)	0.0000	0	0.003067	43	0.932808778	2	2078.99248	1.54	19.44
High	FSDIPNPIGSENSEKTTmP	1	1	1	P02662	M18(Oxidation)	0.0000	0	0.0002668	42	0.812216767	2	2079.95659	1.23	19.76
High	GTQYTDAPSFSDIPNPIGSE	1	1	1	P02662		0.0000	0	1.221E-10	107	2.1708E-07	2	2095.94780	1.07	23.03
High	TDAPSFSDIPNPIGSENSEK	1	1	1	P02662		0.0000	0	5.509E-14	106	2.93577E-07	2	2104.97051	1.65	21.15
High	RHPHPHLSFMAIPPKKNQ	3	1	1	P02668		0.0000	0	1.357E-11	37	4.199944171	5	2135.14661	1.02	16.57
High	YTDAPSFSDIPNPIGSENSE	1	1	1	P02662		0.0000	0	1.40824E-15	116	1.57146E-08	2	2139.93926	1.81	22.84
High	RHPHPHLSFmAIPPKKNQ	2	1	1	P02668	M10(Oxidation)	0.0000	0	0.000001791	27	42.84682153	5	2151.13889	-0.21	15.78
High	DKTEIPTINTIASGEPTSTPT	1	1	1	P02668		0.0000	0	6.159E-09	85	7.71315E-05	2	2173.08891	0.82	20.72
High	MAPKHKEMPFPKYPVEPF	1	1	1	P02666		0.0000	0.006	0.02743	22	176.3318329	3	2173.09897	0.72	19.83
High	VLSRYPSYGLNYYQQKPV	2	1	1	P02668		0.0000	0	0.00003317	75	0.000834969	2	2175.12871	2.46	19.64
High	MAPKHKEmPFPKYPVEPF	1	1	1	P02666	M8(Oxidation)	0.0000	0	0.0001028	21	190.4666551	3	2189.09360	0.58	19.03
High	HKEMPFPKYPVEPFTESQ	6	1	1	P02666		0.0000	0	0.0007974	42	1.180289123	3	2191.05704	1.87	20.09
High	SEEQQQTEDELQDKIHPF	1	1	1	P02666		0.0000	0	0.0001131	34	4.194534125	3	2201.00132	0.88	19.99
High	ARHPHPHLSFMAIPPKKNQ	3	1	1	P02668		0.0000	0	0.000001538	36	4.755476903	4	2206.18715	2.54	16.52
High	HKEmPFPKYPVEPFTESQ	2	1	1	P02666	M4(Oxidation)	0.0000	0	0.007757	33	9.108488273	3	2207.05021	1.06	19.29
High	SDIPNPIGSENSEKTTMPLW	1	1	1	P02662		0.0000	0	0.000000194	53	0.089417473	2	2216.05986	2.61	23.42
High	TKKTKLTEEEKNRLNFLK	1	1	1	P02663		0.0000	0	0.0004409	22	42.4481283	4	2220.27407	1.32	16.62
High	ARHPHPHLSFmAIPPKKNQ	2	1	1	P02668	M11(Oxidation)	0.0000	0	4.063E-07	34	8.870423925	5	2222.17832	0.83	15.74
High	SLSQSKVLPVPQKAVPYPQR	2	1	1	P02666		0.0000	0	6.152E-07	52	0.053199777	3	2222.26792	1.02	18.37
High	QYTDAPSFSDIPNPIGSENSE	1	1	1	P02662		0.0000	0	3.11E-08	69	0.000776118	2	2267.99809	1.82	22.75
High	HKEMPFPKYPVEPFTESQS	1	1	1	P02666		0.0000	0	0.007768	39	2.085552005	3	2278.08652	0.68	20.07
High	HKEmPFPKYPVEPFTESQS	1	1	1	P02666	M4(Oxidation)	0.0000	0	0.0006296	49	0.199191859	2	2294.08232	1.06	19.38
High	QSEEQQQTEDELQDKIHPF	1	1	1	P02666		0.0000	0	0.0001732	38	1.708866216	3	2329.06144	1.49	20.00

High	ARHPHPHLSFmAIPPKKNQD	1	1	1	P02668	M11(Oxidation)	0.0000	0	0.00007616	34	11.17789396	5	2337.20335	-0.03	15.79
High	FFAKESVKDAAGGPGAPADPGRPT	2	2	1	P81265-2		0.0000	0	6.887E-10	58	0.044331055	3	2343.17740	1.91	17.13
High	TQYTDAPSFSDIPNPIGSENSE	1	1	1	P02662		0.0000	0	5.83E-08	62	0.004242968	2	2369.04790	2.64	22.73
High	QYTDAPSFSDIPNPIGSENSEK	1	1	1	P02662		0.0000	0	8.51E-11	98	1.92365E-06	2	2396.09258	1.52	21.62
High	DKTEIPTINTIASGEPTSTPTTE	2	1	1	P02668		0.0000	0	8.459E-08	83	0.00012411	2	2403.17827	0.35	20.68
High	GTQYTDAPSFSDIPNPIGSENSE	4	1	1	P02662		0.0000	0	0.000001154	80	5.87599E-05	2	2426.07012	2.89	22.91
High	QYVLSRYPSYGLNYYQQKPV	1	1	1	P02668		0.0000	0	2.994E-10	42	1.787349547	3	2466.24826	1.22	20.69
High	QDKTEIPTINTIASGEPTSTPTTE	2	1	1	P02668		0.0000	0	0.00001874	71	0.00183246	2	2531.23979	1.50	20.50
High	GTQYTDAPSFSDIPNPIGSENSEK	2	1	1	P02662		0.0000	0	0.0001965	64	0.004476793	2	2554.16142	1.31	21.56
High	DKTEIPTINTIASGEPTSTPTTEAVE	2	1	1	P02668		0.0000	0	0.001607	54	0.097540087	2	2702.33208	2.41	21.16
High	EAYLAEGDEACHAYRGMTKRNRPm	1	0	0		M24(Oxidation)	0.0000	0.002	0.0141	12	691.5812934	5	2785.26289	0.88	14.93
High	AIPPKKNQDKTEIPTINTIASGEPTSTPTTE	1	1	1	P02668		0.0000	0	0.0004476	21	273.418359	4	3279.70009	1.37	19.05
High	ADESTYWGGETTWLGNEVRYSSGNEGHKESGVIDGSESKKGHK	1	0	0			0.0000	0.002	0.01373	11	1114.082893	6	4669.15698	2.27	15.31
